# Identification of *Saccharomyces cerevisiae* Spindle Pole Body Remodeling Factors

**DOI:** 10.1371/journal.pone.0015426

**Published:** 2010-11-12

**Authors:** Kristen B. Greenland, Huiming Ding, Michael Costanzo, Charles Boone, Trisha N. Davis

**Affiliations:** 1 Department of Biochemistry, University of Washington, Seattle, Washington, United States of America; 2 Program in Molecular and Cellular Biology, University of Washington, Seattle, Washington, United States of America; 3 Banting and Best Department of Medical Research, Terrence Donnelly Centre for Cellular and Biomolecular Research, University of Toronto, Toronto, Ontario, Canada; University of Texas-Houston Medical School, United States of America; Ph.D

## Abstract

The *Saccharomyces cerevisiae* centrosome or spindle pole body (SPB) is a dynamic structure that is remodeled in a cell cycle dependent manner. The SPB increases in size late in the cell cycle and during most cell cycle arrests and exchanges components during G1/S. We identified proteins involved in the remodeling process using a strain in which SPB remodeling is conditionally induced. This strain was engineered to express a modified SPB component, Spc110, which can be cleaved upon the induction of a protease. Using a synthetic genetic array analysis, we screened for genes required only when Spc110 cleavage is induced. Candidate SPB remodeling factors fell into several functional categories: mitotic regulators, microtubule motors, protein modification enzymes, and nuclear pore proteins. The involvement of candidate genes in SPB assembly was assessed in three ways: by identifying the presence of a synthetic growth defect when combined with an Spc110 assembly defective mutant, quantifying growth of SPBs during metaphase arrest, and comparing distribution of SPB size during asynchronous growth. These secondary screens identified four genes required for SPB remodeling: *NUP60*, *POM152*, and *NCS2* are required for SPB growth during a mitotic cell cycle arrest, and *UBC4* is required to maintain SPB size during the cell cycle. These findings implicate the nuclear pore, urmylation, and ubiquitination in SPB remodeling and represent novel functions for these genes.

## Introduction

The centrosome is the dominant microtubule-organizing center in mammalian cells and is important for chromosome segregation. Centrosomes facilitate the organization of microtubules during interphase, as well as organizing the bipolar spindle during cell division. Each daughter cell must receive only a single centrosome, and duplication must occur only once during the cell cycle to ensure bipolarity. Centrosome abnormalities, including increased number, size, and microtubule nucleation capacity, are a hallmark of many cancer types, and severity of these defects increases during tumor progression [1]. Although multipolarity is often a consequence of centrosome abnormalities in cancer cells, several studies have shown that the amplified centrosomes coalesce and form a bipolar spindle [1], [2], [3]. This has also been demonstrated in normal cells forced to have a double complement of DNA and centrosomes: retinal pigmented epithelial (RPE1) cells treated with a cytokinesis inhibitor are able to cluster the centrosomes to form a bipolar spindle and proceed through the cell cycle [4]. Minus-end-directed microtubule motor proteins are involved in this clustering process: inhibition of dynein in fibroblasts leads to disassociation of clustered centrosomes [2] and Drosophila kinesin 14 motor protein Ncd is required for focusing of spindle poles [5] and maintaining spindle bipolarity when centrosome amplification is induced [6]. These data demonstrate a cellular response pathway for repairing centrosome and spindle assembly defects.

The spindle pole body (SPB) is the functional equivalent of the mammalian centrosome in *Saccharomyces cerevisiae* and organizes microtubules for chromosome segregation in mitosis and meiosis. The SPB is not a static structure. Instead, the SPB is remodeled in two ways: by growth, in which new components are added, and by exchange, in which old components are replaced by new components. These changes are cell cycle dependent, with growth occurring late in the cell cycle, and exchange occurring around the time of SPB duplication leading to the parent SPB having a mix of old and new components. Cell cycle arrests have various effects on these remodeling phenotypes. When arrested in G1 with α-factor, the SPB core becomes smaller. Conversely, when cells are arrested at metaphase, the SPB core grows. For example, overexpression of Mps1 kinase, which activates the spindle assembly checkpoint, causes SPBs to double in size. Based on the fact that the SPB is remodeled at discrete times during the cell cycle and in response to checkpoint activation, this process is likely to be important for maintenance of the SPB and possibly for assembly of the spindle. [7]

SPB remodeling was observed by tagging the integral SPB component Spc110 with fluorophores and using quantitative fluorescence to determine the level of incorporation or exchange of labeled protein [7]. Determination of the amount of Spc110 using this method is a good measure of the overall SPB core size: comparison of Spc110-YFP fluorescence in tetraploid strains with one to four copies of *SPC110::YFP* showed that SPB fluorescence is proportional to the amount of Spc110-YFP at the pole [7], Spc110 links γ-tubulin to the SPB core and consists of globular domains connected by a long coiled-coil region. Three functional domains have been identified within the protein through mutational analysis for temperature sensitive mutants [8]. The best characterized are *spc110-220*, *spc110-221*, and *spc110-226*, and each of these mutants is defective in one of Spc110′s functions. At the non-permissive temperature, *spc110-221* mutants arrest the cell cycle due to a defect in Spc110-221 attachment to the γ-tubulin complex [8]. Mutant *spc110-226* cells also lose viability at the non-permissive temperature due to a weakened connection, in this case between Spc110-226 and the SPB core [9]. Mutant *spc110-220* contains several point mutations in the calmodulin-binding domain. At the non-permissive temperature, Spc110-220 is not assembled into the pole efficiently due to defective binding of calmodulin [10].

One protein that has been previously shown to affect assembly of SPB components is Mlp2, a nuclear pore-associated protein that binds to SPB core components and affects their assembly into the SPB [11]. Deletion of *MLP2* leads to formation of smaller SPBs, and combining Mlp2 depletion with *spc110-220* exacerbates the assembly defect and is lethal. These data make Mlp2 a likely SPB remodeling factor and implicate nuclear pore proteins in SPB assembly and remodeling.

To identify additional proteins involved in the remodeling process, we developed a system for conditionally inducing SPB remodeling. The remodeling strain contains a version of Spc110 that can be cleaved by TEV protease. Using a synthetic genetic array analysis, we screened for genes required only when cleavage of Spc110 is induced. We hypothesized that remodeling could alleviate SPB damage in these strains either by growth, which could add new Spc110, or by exchange, which could replace damaged Spc110 with functional Spc110. Secondary screens identified four genes required for SPB remodeling. *UBC4* is required to maintain SPB size during the cell cycle, and *NCS2*, *POM152*, and *NUP60* are required for SPB growth during a mitotic cell cycle arrest.

## Results

### Spc110 cleavage causes a Mad1p/Mad2p-dependent cell cycle delay

**Figure 1:**
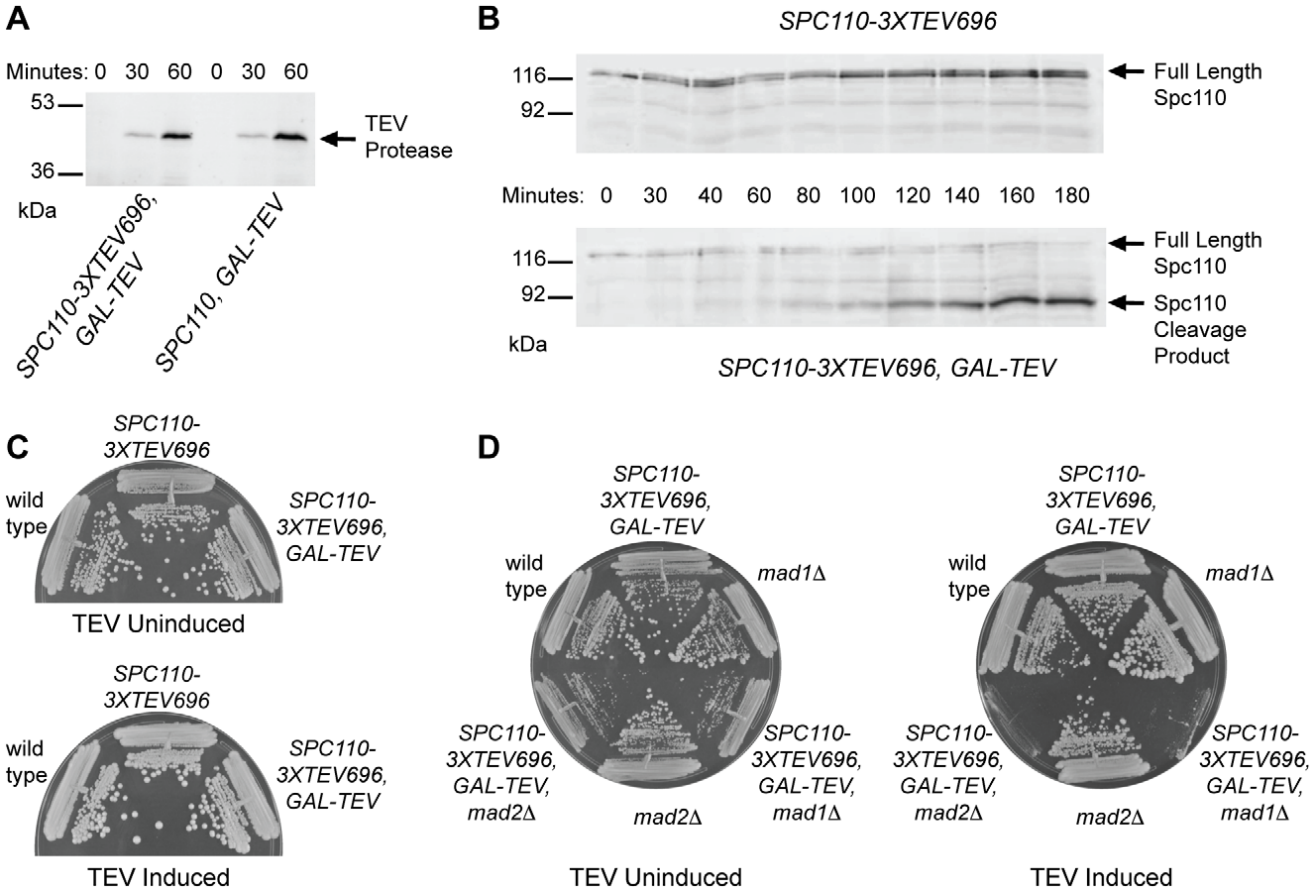
Spc110 cleavage strain phenotype. (A) TEV protease is expressed. A control strain containing galactose-inducible TEV protease and wild type *SPC110* (KGY321-3A) and an Spc110 cleavage strain (KGY57) were grown overnight in YP RAFF liquid media. 2% galactose was added at a cell density of 40 Klett units, and samples were taken for TCA precipitation at 0, 30, and 60 minutes after galactose addition. Protein samples were loaded on a 10% polyacrylamide gel and then analyzed by anti-Myc Western blot for TEV protease. (B) Spc110 is cleaved by TEV protease. A control strain containing cleavable Spc110 but no TEV protease (KGY53) and an Spc110 cleavage strain (KGY61) were grown overnight in YP RAFF liquid media. 2% galactose was added at a cell density of 25 Klett and samples were taken at intervals for TCA precipitation. Protein samples were loaded on 10% polyacrylamide gels and analyzed by anti-Spc110 Western blot. The anti-Spc110 antibody recognizes full length Spc110 and the large cleavage product, which are indicated by arrows. (C) Spc110 cleavage strains are viable on galactose media. Wild type (CRY1), a control strain containing cleavable Spc110 but no TEV protease (KGY54), and an Spc110 cleavage strain (KGY57) were grown on YP RAFF and YP RAFF/GAL plates to determine the growth phenotype of the Spc110 cleavage strain under induced conditions. (D) Spc110 cleavage strain growth is dependent on checkpoint proteins Mad1 and Mad2. Wild type (CRY1), Spc110 cleavage (KGY57), *mad1Δ* (TDY439-1B), Spc110 cleavage + *mad1Δ* (KGY133), *mad2Δ* (SFY127-1A), and Spc110 cleavage + *mad2Δ* (KGY139) strains were grown on YP RAFF and YP RAFF/GAL plates to determine whether the spindle checkpoint is necessary for growth of the Spc110 cleavage strain under induced conditions.

Yeast strains with galactose-inducible Spc110 cleavage were constructed and their phenotypes examined. Galactose-inducible TEV protease was introduced into the genome, along with a tandem array of three TEV cleavage sites in the coiled-coil region of Spc110. Upon induction with galactose, TEV protease is produced (Figure 1A) and cleavage of the majority of Spc110 occurs (Figure 1B). Spc110 cleavage strains form colonies on plates under induced conditions (Figure 1C), indicating that the cells are able to assemble functional SPBs even after Spc110 cleavage. These strains have a longer cell cycle than wild type when Spc110 cleavage is induced: the doubling time for KGY54 (*SPC110-3xTEV696*) is 91 minutes while the doubling time for KGY57 (*SPC110-3xTEV696*, *GAL-TEV*) is 115 minutes. The spindle checkpoint is required to maintain viability of Spc110 cleavage strains under induced conditions (Figure 1D), indicating that a cell cycle delay is necessary to allow for correct spindle assembly in the presence of Spc110 cleavage.

### A genetic screen identifies candidate SPB remodeling factors

To identify additional genes required to survive Spc110 cleavage, we performed a synthetic genetic array (SGA) screen for gene deletions that show a growth defect when combined with Spc110 cleavage. The screen was performed in triplicate and the compiled results are shown in Table S1. Top candidate genes fell into a small number of functional categories: microtubule motors, protein modification enzymes, nuclear pore components, mitotic spindle checkpoint/cell cycle regulators, chromatin remodeling factors, and regulators of mRNA levels (Table 1). Identification of spindle checkpoint genes independently shown to be synthetic lethal with SPB damage (Figure 1D), as well as identification of Spc110 transcriptional regulator *HCM1*, demonstrate the effectiveness of the SGA screen. A subset of candidate genes was chosen for further study and their genetic interaction with Spc110 cleavage was verified in the W303 background to eliminate false positives (Table 1). Deletion of nuclear pore component gene *MLP2*, which was previously shown to have a role in SPB component assembly [11] but was not identified in the SGA screen, did not cause a synthetic growth defect with Spc110 cleavage.

**Table 1:** Genes required for surviving Spc110 cleavage.

Gene	Role	Synthetic Growth Defect in W303^a^
	**Mitosis**	
*CTF19*	Kinetochore	
*CIK1* b	MT motor (kinesin-14 accessory)	Yes
*KAR3* b	MT motor (kinesin-14)	Yes
*VIK1* b	MT motor (kinesin-14 accessory)	Yes
*JNM1* b	MT motor (dynactin complex)	Yes
*DYN3* b	MT motor (dynein inter. light ch.)	Yes
*CLB2*	mitotic cyclin	
*BFA1*	mitotic exit	
*BUB3*	mitotic spindle checkpoint	
*MAD1* b	mitotic spindle checkpoint	Yes
*MAD3*	mitotic spindle checkpoint	
*MAD2* b	mitotic spindle checkpoint	Yes
*CTF18*	sister chromatid cohesion	
*CTF8*	sister chromatid cohesion	
*CHL1*	sister chromatid cohesion	
	**Protein Modification**	
*RTS1* b	PP2A B' subunit	Yes
*NCS2* b	Urmylation	Yes
*PPM1* b	PP2A methyltransferase	No
*UBC4* b	Ubiquitination (E2)	Yes
*UBC7*	Ubiquitination (E2)	
	**Nuclear Pore**	
*SAC3* b	nuclear pore	Yes
*POM152* b	nuclear pore	Yes
*NUP60* b	nuclear pore	Yes
	**Chromatin Remodeling**	
*HTZ1*	Histone	
*SIN3*	Histone deacetylase	
*DOT1* b	Histone methyltransferase	No
*EAF3*	Histone acetyltransferase	
*VPS71*	Part of Swi/Snf remodeling	
	**mRNA Levels**	
*LSM6*	mRNA catabolism	
*NMD2*	mRNA catabolism	
*SKI3*	mRNA catabolism	
*LSM7* b	mRNA catabolism	No
*SKI7*	mRNA catabolism	
*UPF3*	mRNA catabolism	
*PAT1*	mRNA catabolism	
*PUS7*	mRNA splicing	
*HCM1* b	transcriptional activator	Yes
	**Other**	
*SLA1*	Endocytosis	
*YOR052C*	Unknown (zinc-finger protein)	

The top 36 candidates identified in the SGA screen are shown in this table. Deletion of each gene caused decreased growth in the presence of Spc110 cleavage with a p-value <2.17×10^−3^.

a Each of these genes was deleted in the W303 genetic background and the synthetic growth defect verified.

b Chosen for further study.

In addition to systematic false positives, the SGA screen might have identified genes that alter the level of Spc110 or TEV protease. Reduced Spc110 or increased TEV protease could exacerbate the Spc110 cleavage phenotype and cause the cells to die. Spc110 levels were quantified by Western blotting in strains containing a single gene deletion from the subset of candidate genes mentioned above (data not shown). Only *sac3Δ*, *kar3Δ* and *lsm7Δ* caused reduced Spc110 levels, which might account for their appearance in the SGA screen data. TEV protease levels were also quantified by Western blotting in strains containing a single gene deletion and the *GAL-TEV* gene (data not shown). The only gene deletion mutant that had a significant increase in TEV protease production was *ubc4Δ*.

### Deletion mutant crosses to *spc110* temperature sensitive mutants identify candidate SPB remodeling factors with a specific defect in SPB assembly

Deletion of genes involved in assembly should show allele specific defects with *SPC110* mutant *spc110-220*, which was previously determined to be defective in assembly [8]. Each of the candidate gene deletion mutants were mated to a strain carrying *spc110-220* and to strains carrying either of two alleles not involved in assembly: *spc110-221* and *spc110-226* (Figures S1–S16). A summary of the growth phenotypes of these double mutants is compiled in Table 2. Deletion mutants that had the strongest synthetic growth defect in combination with *spc110-220* are *cik1Δ*, *jnm1Δ*, *ncs2Δ*, *ppm1Δ*, *ubc4Δ*, *pom152Δ*, and *nup60Δ*. The allele specific defect with *spc110-220* that was previously shown for mutant *mlp2Δ* [11] was also verified. These candidates are likely to have a role in SPB component assembly based on their specific defect in combination with an assembly mutant.

**Table 2:** The highest temperature that supports normal growth (°C) for haploid progeny of crosses between SPB remodeling candidate gene deletion strains and *spc110* mutants.

Deletions whose strongest effect is on *spc110* assembly mutant *spc110-220*									
	WT	*cik1Δ*	*jnm1Δ*	*ncs2Δ*	*ppm1Δ*	*ubc4Δ*	*pom152Δ*	*nup60Δ*	*mlp2Δ*
*SPC110*	37	25	34	34	32	34	37	37	37
*spc110-220*	32	**<25**	**25**	**<25**	**25**	**25**	**25**	**25**	**25**
*spc110-221*	34	25	34	34	32	**30**	34	**32**	**32**
*spc110-226*	32	25	32	**30**	32	32	32	**30**	32

Deletions that affect all *spc110* mutants									
	WT	*kar3Δ*	*vik1Δ*	*dyn3Δ*	*sac3Δ*	*hcm1Δ*			
*SPC110*	37	25	37	37	34	37			
*spc110-220*	32	**inviable**	**<25**	**30**	**<25**	**<25**			
*spc110-221*	34	**inviable**	**<25**	**30**	**25**	**<25**			
*spc110-226*	32	**<25**	**inviable**	**30**	**25**	**30**			

Deletions that have no effect on *spc110* mutants									
	WT	*rts1Δ*	*dot1Δ*	*lsm7Δ*					
*SPC110*	37	25	37	25					
*spc110-220*	32	25	32	25					
*spc110-221*	34	25	34	25					
*spc110-226*	32	25	32	25					

Results from Figures S1–S16 are compiled in this table: after mating each haploid *spc110* mutant to each haploid deletion mutant, sporulating the diploids, and determining the genotypes of the resulting haploids, controls and double mutants were grown on YPD at several temperatures. The highest temperature that supports normal growth is listed for each haploid *spc110* mutant, each haploid deletion mutant, and double mutant haploids. Double mutants that showed reduced growth compared to both single mutants are highlighted in bold. Normal growth was considered growth comparable to the best growth of the worst growing individual mutant.

### *GAL-MPS1* metaphase arrest identifies candidate SPB remodeling factors with a defect in SPB growth

**Figure 2:**
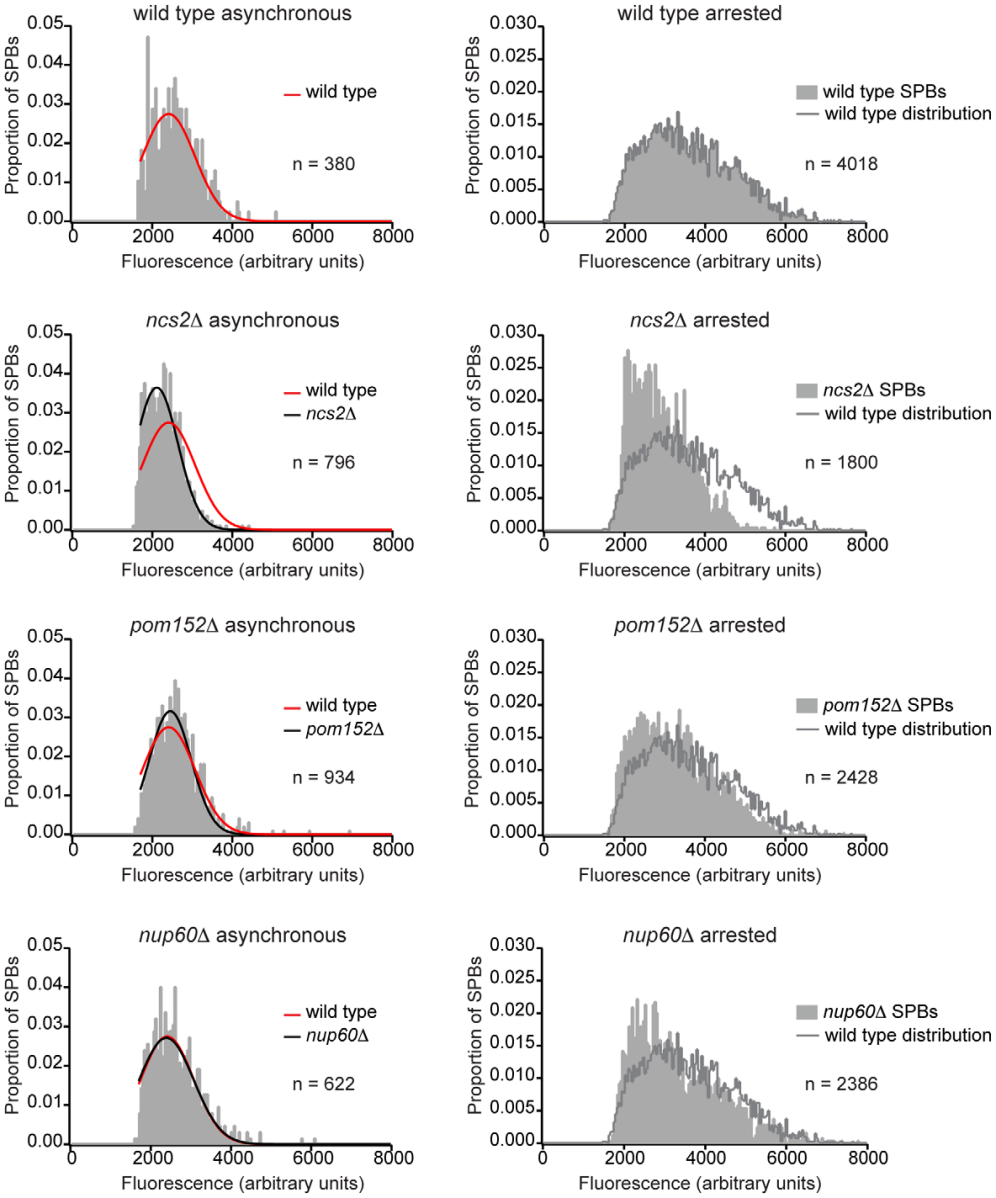
GAL-MPS1 metaphase arrest identifies deletion mutants with a defect in SPB growth. Deletion mutants containing SPC110-GFP and *GAL-MPS1* were grown overnight on YP RAFF plates at room temperature, then struck out onto YP RAFF/GAL plates (time zero). Samples were taken at time zero and fixed for 15 minutes in 3.7% formaldehyde at 30° in a roller drum. Plates were then incubated at room temperature for six hours to induce mitotic arrest (>90% large buds) and samples were taken and fixed in formaldehyde as above for imaging. Fluorescence was quantified for in-focus SPBs and then normalized using the photosensor value of the microscope. The distribution of GAL-MPS1 arrested SPB fluorescence in each mutant strain was compared to the wild type distribution using a Kolmogorov-Smirnov test. *ncs2Δ*, *pom152Δ*, and *nup60Δ* arrested distributions were consistently different from wild type (p-value <1×10∧-5). Histograms of SPB fluorescence values from asynchronously growing (time zero) and metaphase-arrested (six hour time point) yeast from a representative experiment are shown for these strains. The data was normalized by population size and the best fit Gaussian curve was fit to each asynchronous distribution. Histogram bins that fell below the signal to noise cutoff chosen during image analysis were excluded when fitting the Gaussian curves. The wild type asynchronous best fit curve is overlaid on the mutant asynchronous distributions in red for comparison. The wild type arrested SPB fluorescence distribution is overlaid onto the arrested mutant distribution histograms in light grey for comparison.

Six deletion mutants that had a specific growth defect in combination with the *spc110-220* assembly mutant were further characterized for an SPB remodeling phenotype: *jnm1Δ*, *ncs2Δ*, *ppm1Δ*, *ubc4Δ*, *pom152Δ*, and *nup60Δ*. The seventh mutant strain, *cik1Δ*, grows very poorly and was not characterized further. Candidate SPB remodeling gene deletion strains with *SPC110-GFP* and galactose-inducible *MPS1* were imaged during normal asynchronous growth and during a GAL-MPS1 metaphase arrest (>90% large buds). In wild type cells, arresting the cell cycle at metaphase using a GAL-MPS1 arrest causes SPBs to double in size [7]. We therefore quantified the amount of SPB fluorescence and examined the distribution of fluorescence for each strain to identify mutants with an impaired ability to increase SPB size (Figure 2). Cells with *ncs2Δ*, *pom152Δ*, or *nup60Δ* showed a significant defect in SPB growth during metaphase arrest compared to wild type cells (p-value <1×10^−5^ using a Kolmogorov-Smirnov test to compare mutant distributions to wild type in three replicates).

### Deletion of *UBC4*, but not *UBC5*, causes a defect in SPB size regulation

**Figure 3:**
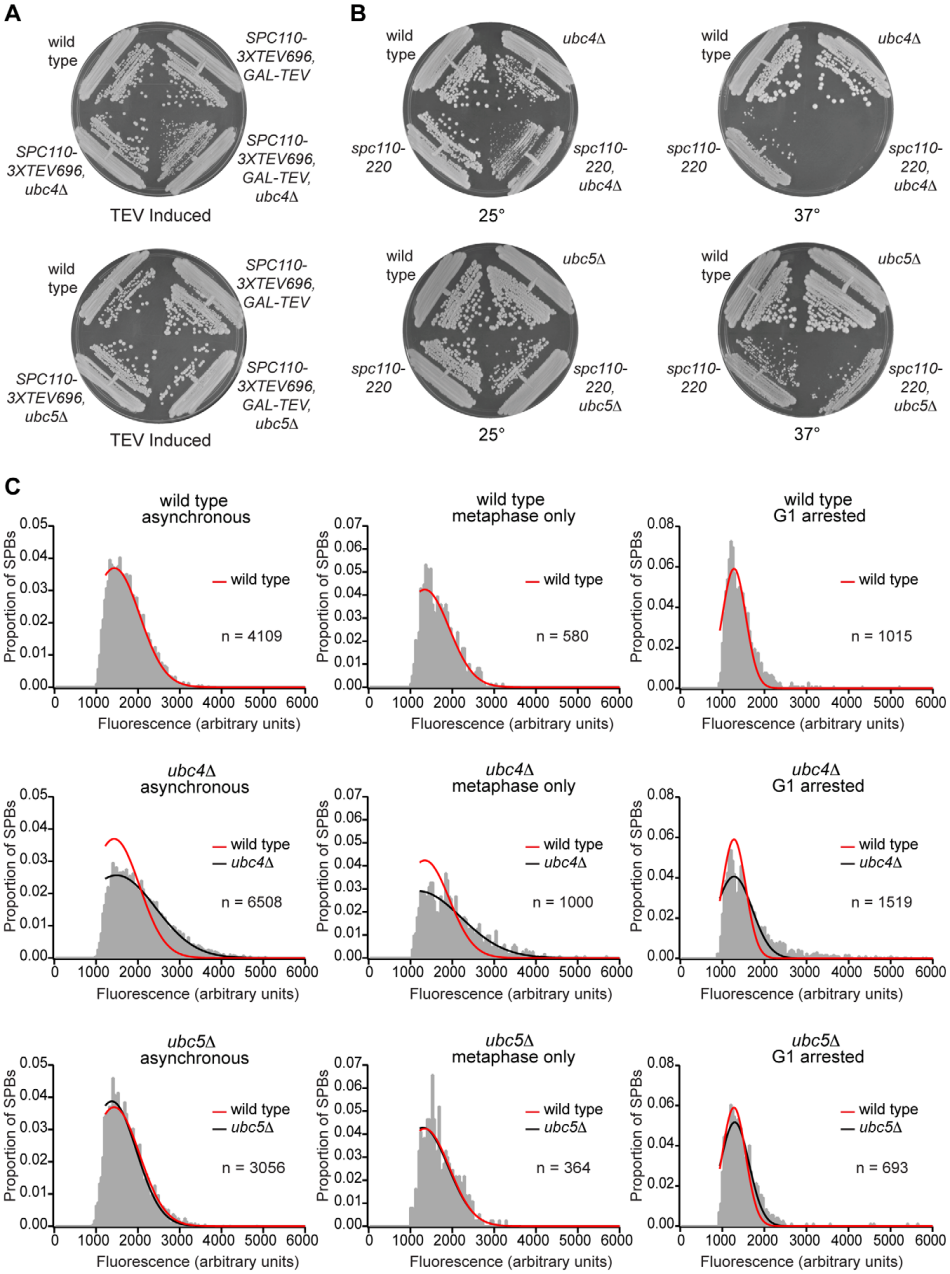
Deletion of *UBC4*, but not *UBC5*, causes a defect in SPB size regulation. (A) *ubc4Δ*, but not *ubc5Δ*, has a synthetic growth defect when combined with Spc110 cleavage. Strains were grown on YP RAFF/GAL plates at room temperature. (B) *ubc4Δ* has a more pronounced growth defect when combined with assembly mutant *spc110-220* than *ubc5Δ*. Strains were grown on YPD plates at 25° and 37°. (C) Asynchronously growing *ubc4Δ*, but not *ubc5Δ*, cells show a defect in SPB size regulation. Strains were grown overnight at 23° in YPD liquid media to a cell density of 25 Klett units. α-factor was added to a concentration of 7.56 µg/ml at time zero and samples were taken and fixed in formaldehyde for imaging of the asynchronous cultures. Strains were then incubated at 23° for 3.5 hours (1.5 generations) and samples were taken from the G1 arrested cells and fixed in formaldehyde for imaging. SPB fluorescence of asynchronously growing and G1 arrested cells was measured and plotted as described. Best fit Gaussian curves were fit to each distribution and the wild type fit is overlaid in red on the *ubc4Δ* and *ubc5Δ* histograms for comparison. SPBs in the asynchronous population that were part of metaphase pairs were isolated and their SPB fluorescence values were also plotted. The distribution of SPB fluorescence after α-factor arrest shows a similar shift in wild type, *ubc4Δ*, and *ubc5Δ* cells, indicating that the mutant strains are able to reduce SPB size in α-factor as well as wild type.

Ubc4 and Ubc5 are ubiquitin-conjugating E2 enzymes that have 77% sequence homology and can functionally complement one another [12]. *UBC4*, but not *UBC5*, was identified in the SGA screen, and the *ubc5Δ* mutant shows no growth defect when combined with Spc110 cleavage (Figure 3A). Combining *spc110-220* with *ubc5Δ* resulted in a milder growth phenotype than with *ubc4Δ* (Figure 3B), and SPBs in the *ubc4Δ* mutant, but not *ubc5Δ*, have a wider fluorescence distribution than wild type during asynchronous growth with a greater number of large SPBs (Figure 3C). The percentage of large SPBs (Spc110::GFP fluorescence value greater than the wild type mean plus one standard deviation) in asynchronously growing wild type cells is 16.1%±0.2% while the percentage of large SPBs in *ubc4Δ* cells is 47.2%±8.7%. Large SPBs in *ubc5Δ* cells make up 13.6%±1.0% of the population, which is similar to the percentage found in wild type SPB populations. A comparable increase in the percentage of large SPBs in *ubc4Δ* cells was seen when SPB core component Spc42 was tagged with GFP (data not shown). The altered distribution of SPB fluorescence seen in *ubc4Δ* cells does not result from an increase in the number of side-by-side SPBs or collapsed spindles because a similar size distribution is seen when the subset of SPBs that are in metaphase pairs is plotted (Figure 3C). Moreover, the *ubc4Δ* mutant does not have an impaired ability to remove core components in G1. Arresting wild-type cells in G1 with α-factor causes a 40% decrease in SPB size [7] and leads to a shift in the peak of fluorescence distribution to lower values (Figure 3C). The *ubc4Δ* mutant shows a shift in peak fluorescence after the arrest that is comparable to wild type (Figure 3C). Asynchronously growing wild type, *ubc4Δ*, and *ubc5Δ* cells were also imaged and the amount of Spc110::GFP or Spc42::GFP was quantified for each strain (Table 3). Mutant *ubc4Δ* SPBs contained high amounts of Spc42::GFP and Spc110::GFP compared to wild type and *ubc5Δ*, which is consistent with *ubc4Δ* cells having a defect in SPB size regulation.

**Table 3:** Mean SPB Fluorescence Comparison for Spc110::GFP and Spc42::GFP in wild type, *ubc4Δ*, and *ubc5Δ* strains.

	Spc110::GFP Fluorescence +/− SD	Spc42::GFP Fluorescence +/− SD
wild type	2470+/−1260	3760+/−2160
*ubc4Δ*	3660+/−2020	5020+/−3570
*ubc5Δ*	2090+/−1340	3870+/−1860

Asynchronously growing cells were imaged and SPB fluorescence was measured for wild type, *ubc4Δ*, and *ubc5Δ* strains. SPB fluorescence was plotted and the mean and standard deviation (SD) were determined by fitting a Gaussian curve to the distribution. Data from a representative experiment is shown. Fluorescence values are in arbitrary units.

## Discussion

SPB remodeling has been shown to occur at discrete times during the cell cycle. However, very little is known about the process of remodeling and the proteins involved in regulating and facilitating SPB growth and component exchange. In this study, we have identified several candidates for involvement in the SPB remodeling process. Proteins identified include microtubule motors, protein modification enzymes, and nuclear pore proteins.

Many of the yeast microtubule motors were identified and had a synthetic growth defect with Spc110 cleavage. This information, coupled with previous studies on motors in other organisms, suggests that motors play a role in assembly of the spindle and specific SPB components. Dynein and Ncd (the Kar3p homolog) have previously been shown to move microtubule bundles to the centrosome in Drosophila [5], and dynein has also been shown to transport pericentrin (the Spc110p homolog) and γ-tubulin to the centrosome in mammalian cells [13]. While deletion of the motors identified in our study did not lead to a defect in SPB growth during metaphase arrest, further characterization of these proteins and their role in SPB remodeling could shed light on the process of spindle assembly.

Our secondary screens identified four proteins that regulate SPB size: Ncs2, Nup60, Pom152, and Ubc4. Loss of Ncs2, Nup60, or Pom152 led to an impaired ability to increase SPB size during metaphase arrest, implicating these proteins in SPB component assembly. Ncs2 is involved in the ubiquitin-related modifier Urm1 pathway and is necessary for thiolation of Lys(UUU) and Glu(UUC) tRNAs [14], [15]. Ncs2 has no known association with SPB proteins. However, our results suggest involvement of the urmylation pathway in regulation of SPB size. Components of the urmylation pathway have been previously shown to have genetic interactions with nuclear pore component *NUP133:* deletion of *URM1* or *UBA4* (Urm1 activator) leads to a synthetic growth defect when combined with *NUP133* deletion [16]. Furthermore, deletion of *NUP133* is synthetic lethal with deletion of another nuclear pore component gene, *NUP60* [17]. We have shown that Nup60 and Pom152 are necessary for surviving Spc110 cleavage and for SPB growth during metaphase arrest. The only protein previously described as having a role in assembly of SPB components is nuclear pore protein Mlp2. We found that *mlp2Δ* does not have a synthetic growth defect when combined with Spc110 cleavage and therefore was not found in our SGA screen. However, attachment of Mlp2 to the nuclear pore is mediated by Nup60 [18]. Pom152 has previously been shown to form a complex with Ncd1 and Pom34 [19], which assembles to form a ring around the nuclear membrane structure of the pore [20]. Additionally, deletion of *POM34* or *POM152* disrupted the function of essential SPB duplication regulator, Mps2 [21]. These data, combined with our data on Ncs2, Nup60, and Pom152, further implicate the nuclear pore in proper assembly of the SPB and suggest that the urmylation pathway may act in conjunction with nuclear pore components to regulate SPB size.

Our screen also identified Ubc4, the ubiquitin-conjugating enzyme (E2). In mammalian cells, tumor suppressor BRCA1 uses a Ubc4 homolog as one of its ubiquitin E2 ligases for conjugating ubiquitin to target proteins [22]. It has also been shown that BRCA1-dependent ubiquitination is important in regulating centrosome number [23], and centrosome amplification is a hallmark of cancer. Our results show that Ubc4 but not its close relative, Ubc5, regulates the size of SPBs during asynchronous growth. Deletion of *UBC4* leads to disruption of SPB size regulation as indicated by increased levels of Spc110 and Spc42 in the poles. Ubiquitination of target proteins by Ubc4 could regulate SPB size by altering levels of SPB proteins or by affecting their incorporation into the pole, thereby changing the nucleation capacity of the SPB. BRCA1 regulates centrosome nucleation activity through ubiquitination of γ-tubulin and a centrosome adaptor component [24], and our data implicate Ubc4 in a conserved centrosome regulation pathway in yeast.

## Materials and Methods

### Media

YPD and SD media was prepared as previously described [25]. YP raffinose plates (YP RAFF) contain 2% raffinose, YP galactose plates and liquid media (YP GAL) contain 2% galactose, and YP raffinose/galactose (YP RAFF/GAL) plates contain 2% raffinose and 2% galactose. YPD NAT plates were made by spreading 30 µl of 10 mg/ml clonNAT (Werner BioAgents, Jena, Germany) solution onto YPD plates. YPD HYG plates are standard YPD supplemented with 0.6 mg/ml hygromycin B. Media used in the SGA screens were made as previously described [26] except where tailored to fit the Spc110 cleavage strains as noted below.

### Plasmids

Plasmids used in this study are listed in Table S2.

#### *SPC110 three TEV protease cleavage site tandem array (SPC110-3xTEV696) plasmids*

QuickChange Site Directed Mutagenesis (Stratagene, La Jolla, CA) was performed on pHS31 [8] to create a *BamH*1 site at *SPC110* base pair 2085 (corresponding to amino acid 696), resulting in plasmid pKG2. DNA oligos with sequence for three TEV cleavage sites and flanking *BamH*1 sites were constructed. Sense and missense oligos (5′-GATCCGAAAATTTATATTTTCAAGGTGAAAATTTATATTTTCAAGGTGAAAATTTATATTTTCAAG-3′ and 5′-GATCCTTGAAAATATAAATTTTCACCTTGAAAATATAAATTTTCACCTTGAAAATATAAATTTTCG-3′) were annealed and ligated to a *BamH*1 digest of pKG2 to create pKG7. *Nco*1 and *Sac*1 digest of pAG25 [27] yielded a fragment containing the nourseothricin (*NAT1*) resistance cassette (natMX4), and this fragment was ligated to the *Nco*1, *Sac*1 large fragment of pFA6a-3HA-kanMX6 plasmid [28] to create pKG9, a plasmid for tagging genes with an HA tag and *NAT1* selectable marker. The HA-natMX4 cassette was amplified from pKG9 and integrated into pKG7 to create a plasmid containing *SPC110-3xTEV696* with an HA tag and a *NAT1* selectable marker (pKG16). This plasmid was then converted to an integrating plasmid by ligating the pRS306 [29] large *AlwN*1 fragment to the pKG16 large *AlwN*1 fragment, resulting in the pKG17 plasmid used for Spc110 cleavage strain construction.

#### *Galactose-inducible TEV protease (GAL-TEV) plasmids*

*GAL-TEV* was amplified by PCR from plasmid 118 (gift from Frank Uhlmann) and ligated into pCR Blunt II-TOPO (Invitrogen Corporation, Carlsbad, CA) to create pKG10. pRS306 was then digested with *Not*1 and *Xho*1 and ligated to the small *Not*1, *Xho*1 fragment of pKG10 to form pKG12, an integrating plasmid containing *GAL-TEV* with a *URA3* marker.

#### *GAL2 plasmid*

Wild type *GAL2* was PCR amplified from HSY2-12C [30] and ligated into pCR Blunt II-TOPO to create pKG11. pRS306 was digested with *Not*1 and *Sac*1 and ligated to the small *Not*1, *Sac*1 fragment of pKG11 to create pKG13, an integrating plasmid containing *GAL2* with a *URA3* marker. QuickChange Site Directed Mutagenesis was performed on pRS315 [29] to create a *BsrG*1 site for subsequent removal of the *LEU2* gene. The *BsrG*1 fragment of the resulting pKG14 plasmid was then ligated into the *BsrG*1 site in pKG13, resulting in plasmid pKG15. The *BsrG*1 site is located in the genomic sequence directly downstream of *GAL2*. pKG15 was checked by restriction digest to ensure the proper orientation.

### Strains

Strains used in this study are listed in Table S3.

#### *Spc110 cleavage strain (W303 background)*

The *SPC110-3xTEV696* fragment was amplified from pKG17 and transformed into GZY7-5B (gift from Gefeng Zhu). Selecting for integrants on YPD NAT plates resulted in strain KGY54, and correct integration of the TEV cleavage sites was confirmed by sequencing. This strain has three TEV protease cleavage sites at amino acid 696 in the only copy of *SPC110*. *GAL-TEV* containing plasmid pKG12 was digested with *Nco*1 for integration at the *URA3* locus and stable integrants were selected for on SD -ura dropout media. The resulting Spc110 cleavage strain is KGY57.

#### *Spc110 cleavage strain for synthetic genetic array (SGA) screening (S288C background)*

Strain Y7029 was transformed with the *Not*1, *Sac*1 fragment of pKG15, which contains wild type *GAL2*. *GAL2* is a plasma membrane galactose permease that is defective in S288C and might be necessary for full activation of galactose-inducible promoters [31]. The resulting strain, KGY39, was then transformed with the *SPC110-3xTEV696* cassette as described in the Spc110 cleavage strain (W303 background) section above to make strain KGY53, and correct integration of the cleavage sites was confirmed by sequencing. The *GAL-TEV::URA3* fragment was amplified from pKG12 and integrated in a region near the *URA3* locus between *TIM9* and *RPR1* because *URA3* and its flanking sequence are deleted in Y7029. Stable integrants were isolated by selection on SD -ura plates resulting in the strain KGY61, which was used in the SGA screens.

#### *Gene deletion strains*

 Gene deletions were made by first PCR amplifying a hygromycin B cassette (hphMX4) from pAG32 [27] using primers that had ends homologous to the flanking DNA of each gene to be deleted. The cassette was then transformed into the diploid strain BSY9 [32] or KGY315. The resulting transformants were dissected and scored for growth on YPD HYG plates. The deletions were checked by PCR to ensure replacement of the target gene with the cassette.

### Synthetic genetic array screen

The SGA screen was performed as previously described [33]. Growth conditions for each step of the screen were as follows. All three Spc110 cleavage strain markers (*SPC110-3XTEV696-HA::natMX4*, *GAL-TEV::URA3*, and *GAL2::LEU2*), as well as the *xΔ::KAN* marker for the deletion, were selected for. Strains were mated on YPD and diploids selected on SD -leu -ura +G418 +NAT media. Diploids were then sporulated and *MAT* **a** *GAL-TEV::URA3* haploids were selected first on SD -his -arg -lys -ura +canavanine +thalysine, then a second round of haploid selection was performed on SD -his -arg -lys -ura -leu +canavanine +thalysine, which selects for cells that are *GAL2::LEU2*. The *xΔ::KAN* haploids were identified by transfer to SD -his -arg -lys -ura -leu +canavanine +thalysine +G418 media, then haploids were transferred to SD -his -arg -lys -ura -leu +canavanine +thalysine +G418 +NAT to identify those containing *SPC110-3XTEV696-HA::natMX4*. Once mutants were identified that contained all of the desired markers, they were transferred to S(galactose) -his -arg -lys -ura -leu +canavanine +thalysine +G418 +NAT and growth on galactose-containing media was compared to growth on SD -his -arg -lys -ura -leu +canavanine +thalysine +G418 +NAT. Genetic interactions from SGA screens were processed and identified as previously described [26].

### Fluorescence microscopy and image analysis

Fluorescently labeled strains were mounted on a 1% agarose in S media pad and SPBs were imaged using a DeltaVision Core Restoration Microscopy System (Applied Precision, Issaquah, WA) that incorporates an Olympus U-plan Apo 100X oil objective (NA, 1.35). GFP filter sets (ex. 470/40, em. 525/50) were from Chroma Technology. Images were captured using a Photometrics Coolsnap HQ camera (Photometrics, Pleasanton, CA) and analyzed using the Fluorcal software program [34] to identify SPBs that were in focus in a single focal plane. Fluorescence intensity was calculated by determining the integrated intensity in a 5×5 pixel square around each SPB and subtracting background fluorescence.

### Analysis of SPB fluorescence data

Histograms of SPB fluorescence intensity values were constructed and Gaussian curves were fit to the data using Igor Pro version 6.12 (WaveMetrics, Portland, OR). Kolmogorov-Smirnov tests were performed using the KS version 2.0 for NeuroMatic version 2.00 Igor Pro procedure.

## Supporting Information

Table S1 **Synthetic genetic array (SGA) screen data.**
(DOC)

Table S2 **Plasmids used in this study.**
(DOC)

Table S3 **Yeast strains used in this study.**
(DOC)

Figure S1 ***spc110*** **mutants crossed with** ***cik1*** **.** Haploids with the genotypes indicated on the left were grown on YPD plates and incubated at the temperatures indicated.
(TIF)

Figure S2 ***spc110*** **mutants crossed with** ***kar3*** **.** Haploids with the genotypes indicated on the left were grown on YPD plates and incubated at the temperatures indicated.
(TIF)

Figure S3 ***spc110*** **mutants crossed with** ***vik1*** **.** Haploids with the genotypes indicated on the left were grown on YPD plates and incubated at the temperatures indicated.
(TIF)

Figure S4 ***spc110*** **mutants crossed with** ***jnm1*** **.** Haploids with the genotypes indicated on the left were grown on YPD plates and incubated at the temperatures indicated.
(TIF)

Figure S5 ***spc110*** **mutants crossed with** ***dyn3*** **.** Haploids with the genotypes indicated on the left were grown on YPD plates and incubated at the temperatures indicated.
(TIF)

Figure S6 ***spc110*** **mutants crossed with** ***rts1*** **.** Haploids with the genotypes indicated on the left were grown on YPD plates and incubated at the temperatures indicated.
(TIF)

Figure S7 ***spc110*** **mutants crossed with** ***ncs2*** **.** Haploids with the genotypes indicated on the left were grown on YPD plates and incubated at the temperatures indicated.
(TIF)

Figure S8 ***spc110*** **mutants crossed with** ***ppm1*** **.** Haploids with the genotypes indicated on the left were grown on YPD plates and incubated at the temperatures indicated.
(TIF)

Figure S9 ***spc110*** **mutants crossed with** ***ubc4*** **.** Haploids with the genotypes indicated on the left were grown on YPD plates and incubated at the temperatures indicated.
(TIF)

Figure S10 ***spc110*** **mutants crossed with** ***sac3*** **.** Haploids with the genotypes indicated on the left were grown on YPD plates and incubated at the temperatures indicated.
(TIF)

Figure S11 ***spc110*** **mutants crossed with** ***pom152*** **.** Haploids with the genotypes indicated on the left were grown on YPD plates and incubated at the temperatures indicated.
(TIF)

Figure S12 ***spc110*** **mutants crossed with** ***nup60*** **.** Haploids with the genotypes indicated on the left were grown on YPD plates and incubated at the temperatures indicated.
(TIF)

Figure S13 ***spc110*** **mutants crossed with** ***mlp2*** **.** Haploids with the genotypes indicated on the left were grown on YPD plates and incubated at the temperatures indicated.
(TIF)

Figure S14 ***spc110*** **mutants crossed with** ***dot1*** **.** Haploids with the genotypes indicated on the left were grown on YPD plates and incubated at the temperatures indicated.
(TIF)

Figure S15 ***spc110*** **mutants crossed with** ***lsm7*** **.** Haploids with the genotypes indicated on the left were grown on YPD plates and incubated at the temperatures indicated.
(TIF)

Figure S16 ***spc110*** **mutants crossed with** ***hcm1*** **.** Haploids with the genotypes indicated on the left were grown on YPD plates and incubated at the temperatures indicated.
(TIF)
