# Supplementary material for: Identification of Saccharomyces cerevisiae Spindle Pole Body Remodeling Factors
Source: PLoS One. 2010 Nov 12;5(11):e15426. doi: 10.1371/journal.pone.0015426 (PMC2980476; doi:10.1371/journal.pone.0015426)
Supplement: Table S1 — Synthetic genetic array (SGA) screen data. (DOC) [file pone.0015426.s001.doc]

**Table S1. Synthetic genetic array (SGA) screen data^a^.**

| **Gene** | **p-Value**  **x 10^-3^** | **Con**  **Mean^b^** | **Con**  **Dev^c^** | **Exp**  **Mean^d^** | **Exp**  **Dev^e^** | **Diff**  **Mean^f^** |
| --- | --- | --- | --- | --- | --- | --- |
| MAD1 | 2.17 | 1.09 | 0.105 | 0.000331 | 0.000741 | 1.09 |
| MAD3 | 2.17 | 1.03 | 0.0753 | 0.0561 | 0.0540 | 0.978 |
| MAD2 | 2.17 | 1.04 | 0.0850 | 0.0765 | 0.155 | 0.964 |
| SAC3 | 2.17 | 1.03 | 0.123 | 0.0808 | 0.129 | 0.951 |
| CIK1 | 2.17 | 0.921 | 0.115 | 0.00449 | 0.0100 | 0.916 |
| KAR3 | 2.17 | 0.823 | 0.0401 | 0 | 0 | 0.823 |
| CTF18 | 2.17 | 0.728 | 0.0833 | 0.0104 | 0.0110 | 0.717 |
| LSM6 | 2.17 | 1.20 | 0.101 | 0.526 | 0.361 | 0.679 |
| CTF8 | 2.17 | 0.853 | 0.0642 | 0.179 | 0.115 | 0.674 |
| VIK1 | 2.17 | 1.06 | 0.0471 | 0.468 | 0.129 | 0.596 |
| BFA1 | 2.17 | 1.00 | 0.0969 | 0.429 | 0.117 | 0.573 |
| HTZ1 | 2.17 | 0.711 | 0.177 | 0.138 | 0.0798 | 0.573 |
| DOT1 | 2.17 | 0.887 | 0.116 | 0.332 | 0.138 | 0.555 |
| RTS1 | 2.17 | 0.565 | 0.148 | 0.0472 | 0.0843 | 0.518 |
| LSM7 | 2.17 | 1.25 | 0.110 | 0.756 | 0.226 | 0.490 |
| NMD2 | 2.17 | 1.09 | 0.155 | 0.638 | 0.180 | 0.456 |
| HCM1 | 2.17 | 0.864 | 0.0999 | 0.410 | 0.145 | 0.454 |
| NCS2 | 2.17 | 0.809 | 0.0887 | 0.358 | 0.227 | 0.451 |
| CHL1 | 2.17 | 1.05 | 0.113 | 0.613 | 0.0857 | 0.433 |
| SKI7 | 2.17 | 1.13 | 0.0814 | 0.697 | 0.117 | 0.432 |
| BUB3 | 2.17 | 0.425 | 0.163 | 0 | 0 | 0.425 |
| CLB2 | 2.17 | 0.994 | 0.0978 | 0.576 | 0.110 | 0.418 |
| POM152 | 2.17 | 0.966 | 0.0271 | 0.563 | 0.149 | 0.403 |
| SKI3 | 2.17 | 0.987 | 0.0418 | 0.589 | 0.126 | 0.398 |
| YOR052C | 2.17 | 1.07 | 0.0692 | 0.693 | 0.0686 | 0.382 |
| NUP60 | 2.17 | 1.01 | 0.0571 | 0.633 | 0.121 | 0.374 |
| EAF3 | 2.17 | 1.02 | 0.0442 | 0.643 | 0.0829 | 0.372 |
| UPF3 | 2.17 | 1.06 | 0.0855 | 0.701 | 0.0885 | 0.358 |
| PAT1 | 2.17 | 1.32 | 0.117 | 0.970 | 0.151 | 0.353 |
| VPS71 | 2.17 | 0.894 | 0.122 | 0.545 | 0.0972 | 0.349 |
| CTF19 | 2.17 | 1.13 | 0.0653 | 0.787 | 0.0688 | 0.344 |
| JNM1 | 2.17 | 0.850 | 0.0532 | 0.539 | 0.111 | 0.311 |
| DYN3 | 2.17 | 0.926 | 0.0277 | 0.616 | 0.0832 | 0.310 |
| UBC4 | 2.17 | 0.988 | 0.0918 | 0.693 | 0.0926 | 0.295 |
| SLA1 | 2.17 | 0.819 | 0.0830 | 0.533 | 0.0690 | 0.286 |
| PPM1 | 2.17 | 0.800 | 0.0944 | 0.525 | 0.0240 | 0.275 |
| PUS7 | 2.17 | 0.936 | 0.0315 | 0.671 | 0.136 | 0.266 |
| UBC7 | 2.17 | 1.11 | 0.0879 | 0.876 | 0.0723 | 0.233 |
| SIN3 | 2.17 | 0.266 | 0.0545 | 0.0660 | 0.0483 | 0.200 |
| SAM37 | 4.33 | 0.755 | 0.221 | 0.0918 | 0.153 | 0.663 |
| IKS1 | 4.33 | 1.12 | 0.0818 | 0.783 | 0.173 | 0.336 |
| SNF3 | 4.33 | 0.798 | 0.0723 | 0.475 | 0.162 | 0.323 |
| YIL161W | 4.33 | 0.958 | 0.134 | 0.639 | 0.0935 | 0.318 |
| RRD2 | 4.33 | 0.906 | 0.0712 | 0.640 | 0.131 | 0.265 |
| RPL16B | 4.33 | 0.982 | 0.0360 | 0.733 | 0.169 | 0.249 |
| CTF3 | 4.33 | 1.04 | 0.0481 | 0.810 | 0.0987 | 0.225 |
| MMS2 | 6.49 | 0.950 | 0.143 | 0.586 | 0.154 | 0.364 |
| SKI2 | 6.49 | 0.843 | 0.116 | 0.523 | 0.0931 | 0.320 |
| YGR122W | 6.49 | 0.456 | 0.132 | 0.168 | 0.0849 | 0.289 |
| MSA1 | 6.49 | 0.978 | 0.0509 | 0.696 | 0.202 | 0.282 |
| PDR16 | 6.49 | 1.05 | 0.0580 | 0.810 | 0.124 | 0.241 |
| BUB2 | 8.66 | 0.994 | 0.141 | 0.516 | 0.235 | 0.478 |
| SLK19 | 8.66 | 0.927 | 0.140 | 0.546 | 0.183 | 0.381 |
| PSD1 | 8.66 | 0.693 | 0.114 | 0.403 | 0.120 | 0.290 |
| ASE1 | 8.66 | 1.10 | 0.0924 | 0.814 | 0.145 | 0.288 |
| LAT1 | 8.66 | 0.707 | 0.0850 | 0.435 | 0.119 | 0.272 |
| FAP1 | 10.8 | 0.946 | 0.136 | 0.644 | 0.139 | 0.302 |
| UBC13 | 10.8 | 1.00 | 0.0944 | 0.781 | 0.140 | 0.221 |
| NUP2 | 10.8 | 0.990 | 0.0697 | 0.775 | 0.111 | 0.215 |
| SSE1 | 13.0 | 0.805 | 0.209 | 0.324 | 0.225 | 0.482 |
| PPQ1 | 13.0 | 0.780 | 0.146 | 0.459 | 0.156 | 0.321 |
| EAF7 | 13.0 | 0.651 | 0.148 | 0.341 | 0.117 | 0.310 |
| SWC3 | 13.0 | 0.782 | 0.0721 | 0.546 | 0.161 | 0.236 |
| YNL120C | 13.0 | 0.704 | 0.113 | 0.484 | 0.0748 | 0.220 |
| YJL169W | 15.2 | 0.916 | 0.124 | 0.668 | 0.117 | 0.248 |
| EPS1 | 17.3 | 0.930 | 0.0398 | 0.637 | 0.227 | 0.293 |
| OCA4 | 17.3 | 1.01 | 0.142 | 0.750 | 0.0593 | 0.258 |
| FLD1 | 17.3 | 1.02 | 0.0476 | 0.816 | 0.145 | 0.205 |
| DYN1 | 17.3 | 0.957 | 0.0656 | 0.799 | 0.105 | 0.158 |
| COX5A | 19.5 | 0.728 | 0.0369 | 0.288 | 0.287 | 0.440 |
| YGL217C | 19.5 | 1.06 | 0.118 | 0.775 | 0.167 | 0.287 |
| DEP1 | 19.5 | 0.514 | 0.172 | 0.251 | 0.0955 | 0.263 |
| TUB3 | 19.5 | 1.07 | 0.0704 | 0.891 | 0.109 | 0.178 |
| CIN4 | 19.5 | 0.998 | 0.0327 | 0.856 | 0.0906 | 0.142 |
| SWC5 | 21.6 | 0.614 | 0.179 | 0.295 | 0.169 | 0.319 |
| MRS2 | 21.6 | 0.602 | 0.0881 | 0.318 | 0.168 | 0.284 |
| EBS1 | 23.8 | 0.947 | 0.0598 | 0.709 | 0.173 | 0.238 |
| UBP2 | 23.8 | 0.851 | 0.0867 | 0.620 | 0.143 | 0.230 |
| KAR9 | 26.0 | 0.959 | 0.0735 | 0.826 | 0.0816 | 0.133 |
| KIP3 | 28.1 | 1.03 | 0.0599 | 0.830 | 0.149 | 0.199 |
| TIR3 | 32.5 | 1.14 | 0.0100 | 0.664 | 0.471 | 0.477 |
| INO2 | 32.5 | 0.740 | 0.164 | 0.448 | 0.170 | 0.292 |
| PDA1 | 32.5 | 0.645 | 0.106 | 0.398 | 0.174 | 0.247 |
| PPE1 | 39.0 | 0.948 | 0.139 | 0.674 | 0.193 | 0.273 |
| PAP2 | 41.1 | 0.867 | 0.109 | 0.381 | 0.383 | 0.486 |
| YGL214W | 41.1 | 0.817 | 0.168 | 0.578 | 0.133 | 0.239 |
| PAC1 | 43.3 | 0.923 | 0.146 | 0.666 | 0.164 | 0.257 |
| HOG1 | 43.3 | 0.945 | 0.0974 | 0.708 | 0.200 | 0.237 |
| LGE1 | 47.6 | 0.570 | 0.253 | 0.234 | 0.151 | 0.336 |
| YDJ1 | 49.8 | 0.899 | 0.142 | 0.582 | 0.252 | 0.317 |
| PHO88 | 56.3 | 0.922 | 0.114 | 0.394 | 0.438 | 0.528 |
| YER119C-A | 58.4 | 0.476 | 0.247 | 0.145 | 0.184 | 0.330 |
| BNI1 | 60.6 | 0.573 | 0.409 | 0 | 0 | 0.573 |
| HRT3 | 60.6 | 0.846 | 0.154 | 0.364 | 0.391 | 0.481 |
| ARP6 | 60.6 | 0.357 | 0.336 | 0 | 0 | 0.357 |
| UME6 | 60.6 | 0.793 | 0.159 | 0.460 | 0.279 | 0.334 |
| BUB1 | 60.6 | 0.311 | 0.259 | 0 | 0 | 0.311 |
| RCO1 | 67.1 | 0.960 | 0.106 | 0.685 | 0.254 | 0.274 |
| INO4 | 71.4 | 0.682 | 0.187 | 0.404 | 0.200 | 0.278 |
| PAC10 | 82.3 | 0.827 | 0.125 | 0.478 | 0.320 | 0.349 |
| YNL171C | 88.7 | 0.517 | 0.259 | 0.162 | 0.231 | 0.355 |
| YLR072W | 106 | 1.02 | 0.113 | 0.497 | 0.515 | 0.521 |

^a^Linkage groups for *SPC110*, *URA3*, *CAN1*, and *LYP1* have been removed. The data has been sorted by p-value then differential mean. Genetic interactions from SGA screens were processed and identified as previously described [1].

^b^Con Mean: mean colony size on the control media (SD media).

^c^Con Dev: standard deviation for the control mean.

^d^Exp Mean: mean colony size on the experimental media (S media with galactose).

^e^Exp Dev: standard deviation for the experimental mean.

^f^Diff Mean: difference between the mean size of the control colony and the mean size of the experimental colony.

References

1. Tong AH, Lesage G, Bader GD, Ding H, Xu H, et al. (2004) Global mapping of the yeast genetic interaction network. Science 303: 808-813.
