# Supplementary material for: Identification of Saccharomyces cerevisiae Spindle Pole Body Remodeling Factors
Source: PLoS One. 2010 Nov 12;5(11):e15426. doi: 10.1371/journal.pone.0015426 (PMC2980476; doi:10.1371/journal.pone.0015426)
Supplement: Table S2 — Plasmids used in this study. (DOC) [file pone.0015426.s002.doc]

**Table S2. Plasmids used in this study.**

| **Plasmid** | **Genotype** | **Source** |
| --- | --- | --- |
| 118 | Ylp204 with GAL-NLS-myc9-TEVprotease-NLS2::TRP1 | Frank Uhlmann |
| pAG25 | natMX4 | [1] |
| pAG32 | hphMX4 | [2] |
| pCR Blunt II-TOPO | LacZα f1 origin KAN | Invitrogen |
| pFA6a-3HA-kanMX6 | 3xHA kanMX6 | [3] |
| pHS26 | 2μm SPC110 LYS2 ADE3 | [4] |
| pHS31 | CEN6 ARSH4 URA3 SPC110 | [5] |
| pKG2 | SPC110 with BamH1 site at aa 696 URA3 | This Study |
| pKG7 | SPC110-3xTEV696 URA3 | This Study |
| pKG9 | 3xHA natMX4 | This Study |
| pKG10 | LacZα f1 origin KAN GAL-TEV | This Study |
| pKG11 | LacZα f1 origin KAN GAL2 | This Study |
| pKG12 | pRS306 with GAL-TEV | This Study |
| pKG13 | pRS306 with GAL2 | This Study |
| pKG14 | pRS315 with BsrG1 site | This Study |
| pKG15 | GAL2 LEU2 URA3 | This Study |
| pKG16 | SPC110-3xTEV696-3xHA::natMX4 URA3 | This Study |
| pKG17 | pRS306 with SPC110-3xTEV696-3xHA::natMX4 | This Study |
| pRS306 | URA3 f1 origin | [6] |
| pRS315 | CEN6 ARSH4 LEU2 f1 origin | [6] |

References

1. Hazbun TR, Malmstrom L, Anderson S, Graczyk BJ, Fox B, et al. (2003) Assigning function to yeast proteins by integration of technologies. Mol Cell 12: 1353-1365.

2. Goldstein AL, McCusker JH (1999) Three new dominant drug resistance cassettes for gene disruption in Saccharomyces cerevisiae. Yeast 15: 1541-1553.

3. Bahler J, Wu JQ, Longtine MS, Shah NG, McKenzie A, 3rd, et al. (1998) Heterologous modules for efficient and versatile PCR-based gene targeting in Schizosaccharomyces pombe. Yeast 14: 943-951.

4. Sundberg HA, Goetsch L, Byers B, Davis TN (1996) Role of calmodulin and Spc110p interaction in the proper assembly of spindle pole body compenents. J Cell Biol 133: 111-124.

5. Sundberg HA, Davis TN (1997) A mutational analysis identifies three functional regions of the spindle pole component Spc110p in Saccharomyces cerevisiae. Mol Biol Cell 8: 2575-2590.

6. Sikorski RS, Hieter P (1989) A system of shuttle vectors and yeast host strains designed for efficient manipulation of DNA in Saccharomyces cerevisiae. Genetics 122: 19-27.
