# Supplementary material for: Identification of Saccharomyces cerevisiae Spindle Pole Body Remodeling Factors
Source: PLoS One. 2010 Nov 12;5(11):e15426. doi: 10.1371/journal.pone.0015426 (PMC2980476; doi:10.1371/journal.pone.0015426)
Supplement: Table S3 — Yeast strains used in this study. (DOC) [file pone.0015426.s003.doc]

**Table S3. Yeast strains used in this study^a^.**

| **Strain** | **Genotype** | **Source** |
| --- | --- | --- |
| BSY9 | *MAT****a****/MATα ADE3/ade3Δ CYH2^s^/cyh2^r^* | [1] |
| CRY1 | *MAT****a*** | Robert Fuller |
| GZY7-5B | *MAT****a*** *ade3Δ-100 cyh2^r^ lys2Δ::HIS3* | Gefeng Zhu |
| HSY2-12C | *MAT****a*** *ade3Δ lys2Δ::HIS3 spc110Δ::TRP1 pHS26* | [2] |
| HSY11-3B | *MATα spc110-220* | [3] |
| HSY11-4D | *MAT****a*** *ade3Δ HIS3 spc110-220* | [3] |
| HSY18-18A | *MATα spc110-221* | [4] |
| HSY18-27A | *MAT****a*** *ade3Δ spc110-221* | [4] |
| KGY39 | *MATα can1Δ::STE2pr-HIS3 lyp1Δ his3Δ1 leu2Δ0 ura3Δ0 met15Δ0 LYS2 GAL2::LEU2 (S288C background)* | This Study |
| KGY53 | *MATα can1Δ::STE2pr-HIS3 lyp1Δ his3Δ1 leu2Δ0 ura3Δ0 met15Δ0 LYS2 GAL2::LEU2 SPC110-3xTEV696-HA::natMX4 (S288C background)* | This Study |
| KGY54 | *MAT****a*** *ade3Δ-100 cyh2^r^ lys2Δ::HIS3 SPC110-3xTEV696-HA::natMX4* | This Study |
| KGY57 | *MAT****a*** *ade3Δ-100 cyh2^r^ lys2Δ::HIS3 SPC110-3XTEV696-HA::natMX4 GAL-NLS-myc9-TEVprotease-NLS2::URA3* | This Study |
| KGY61 | *MATα can1Δ::STE2pr-HIS3 lyp1Δ his3Δ1 leu2Δ0 ura3Δ0 met15Δ0 LYS2 GAL2::LEU2 SPC110-3XTEV696-HA::natMX4 GAL-NLS-myc9-TEVprotease-NLS2::URA3 (S288C background)* | This Study |
| KGY133-2C | *MAT****a*** *ade3Δ SPC110-3XTEV696-HA::natMX4 GAL-NLS-myc9-TEVprotease-NLS2::URA3 mad1Δ::URA3* | This Study |
| KGY139-1D | *MATα* *ade3Δ-100 cyh2^r^ lys2Δ::HIS3 SPC110-3XTEV696-HA::natMX4 GAL-NLS-myc9-TEVprotease-NLS2::URA3 mad2Δ::kanMX6* | This Study |
| KGY170-2A | *MAT****a*** *sac3Δ::hphMX4* | This Study |
| KGY171-2D | *MATα nup60Δ::hphMX4* | This Study |
| KGY172-1D | *MATα ade3Δ dyn3Δ::hphMX4* | This Study |
| KGY173-1A | *MAT****a*** *ade3Δ kar3Δ::hphMX4* | This Study |
| KGY174-13B | *MATα ade3Δ hcm1Δ::hphMX4* | This Study |
| KGY175-8B | *MAT****a*** *ade3Δ rts1Δ::hphMX4* | This Study |
| KGY177-9C | *MAT****a*** *ade3Δ jnm1Δ::hphMX4* | This Study |
| KGY178-2C | *MAT****a*** *ade3Δ dot1Δ::hphMX4* | This Study |
| KGY179-3B | *MAT****a*** *ade3Δ pom152Δ::hphMX4* | This Study |
| KGY182 | *MAT****a****/MATα SAC3/sac3Δ::hphMX4 SPC110/spc110-220* | This Study |
| KGY183 | *MAT****a****/MATα ADE3/ade3Δ HIS3/his3-11,15 NUP60/nup60Δ::hphMX4 SPC110/spc110-220* | This Study |
| KGY184 | *MAT****a****/MATα ade3Δ/ade3Δ HIS3/his3-11,15 DYN3/dyn3Δ::hphMX4 SPC110/spc110-220* | This Study |
| KGY185 | *MAT****a****/MATα ADE3/ade3Δ KAR3/kar3Δ::hphMX4 SPC110/spc110-220* | This Study |
| KGY186 | *MAT****a****/MATα ade3Δ/ade3Δ HIS3/his3-11,15 HCM1/hcm1Δ::hphMX4 SPC110/spc110-220* | This Study |
| KGY187 | *MAT****a****/MATα ADE3/ade3Δ RTS1/rts1Δ::hphMX4 SPC110/spc110-220* | This Study |
| KGY189 | *MAT****a****/MATα ADE3/ade3Δ JNM1/jnm1Δ::hphMX4 SPC110/spc110-220* | This Study |
| KGY190 | *MAT****a****/MATα ADE3/ade3Δ DOT1/dot1Δ::hphMX4 SPC110/spc110-220* | This Study |
| KGY191 | *MAT****a****/MATα ADE3/ade3Δ POM152/pom152Δ::hphMX4 SPC110/spc110-220* | This Study |
| KGY192 | *MAT****a****/MATα ADE3/ade3Δ HIS3/his3-11,15 UBC4/ubc4Δ::hphMX4 SPC110/spc110-220* | This Study |
| KGY195 | *MAT****a****/MATα SAC3/sac3Δ::hphMX4 SPC110/spc110-221* | This Study |
| KGY196 | *MAT****a****/MATα ADE3/ade3Δ NUP60/nup60Δ::hphMX4 SPC110/spc110-221* | This Study |
| KGY197 | *MAT****a****/MATα ade3Δ/ade3Δ DYN3/dyn3Δ::hphMX4 SPC110/spc110-221* | This Study |
| KGY198 | *MAT****a****/MATα ADE3/ade3Δ KAR3/kar3Δ::hphMX4 SPC110/spc110-221* | This Study |
| KGY199 | *MAT****a****/MATα ade3Δ/ade3Δ HCM1/hcm1Δ::hphMX4 SPC110/spc110-221* | This Study |
| KGY200 | *MAT****a****/MATα ADE3/ade3Δ RTS1/rts1Δ::hphMX4 SPC110/spc110-221* | This Study |
| KGY202 | *MAT****a****/MATα ADE3/ade3Δ JNM1/jnm1Δ::hphMX4 SPC110/spc110-221* | This Study |
| KGY203 | *MAT****a****/MATα ADE3/ade3Δ DOT1/dot1Δ::hphMX4 SPC110/spc110-221* | This Study |
| KGY204 | *MAT****a****/MATα ADE3/ade3Δ POM152/pom152Δ::hphMX4 SPC110/spc110-221* | This Study |
| KGY205 | *MAT****a****/MATα ADE3/ade3Δ UBC4/ubc4Δ::hphMX4 SPC110/spc110-221* | This Study |
| KGY208 | *MAT****a****/MATα ADE3/ade3Δ LYS2/lys2Δ::HIS3 SAC3/sac3Δ::hphMX4 SPC110/spc110-226* | This Study |
| KGY209 | *MAT****a****/MATα ADE3/ade3Δ LYS2/lys2Δ::HIS3 NUP60/nup60Δ::hphMX4 SPC110/spc110-226* | This Study |
| KGY210 | *MAT****a****/MATα ade3Δ/ade3Δ LYS2/lys2Δ::HIS3 DYN3/dyn3Δ::hphMX4 SPC110/spc110-226* | This Study |
| KGY211 | *MAT****a****/MATα ade3Δ/ade3Δ LYS2/lys2Δ::HIS3 KAR3/kar3Δ::hphMX4 SPC110/spc110-226* | This Study |
| KGY212 | *MAT****a****/MATα ade3Δ/ade3Δ LYS2/lys2Δ::HIS3 HCM1/hcm1Δ::hphMX4 SPC110/spc110-226* | This Study |
| KGY213 | *MAT****a****/MATα ade3Δ/ade3Δ LYS2/lys2Δ::HIS3 RTS1/rts1Δ::hphMX4 SPC110/spc110-226* | This Study |
| KGY215 | *MAT****a****/MATα ade3Δ/ade3Δ LYS2/lys2Δ::HIS3 JNM1/jnm1Δ::hphMX4 SPC110/spc110-226* | This Study |
| KGY216 | *MAT****a****/MATα ade3Δ/ade3Δ LYS2/lys2Δ::HIS3 DOT1/dot1Δ::hphMX4 SPC110/spc110-226* | This Study |
| KGY217 | *MAT****a****/MATα ade3Δ/ade3Δ LYS2/lys2Δ::HIS3 POM152/pom152Δ::hphMX4 SPC110/spc110-226* | This Study |
| KGY218 | *MAT****a****/MATα ADE3/ade3Δ LYS2/lys2Δ::HIS3 UBC4/ubc4Δ::hphMX4 SPC110/spc110-226* | This Study |
| KGY245 | *MAT****a****/MATα ade3Δ/ade3Δ-100 LYS2/lys2Δ::HIS3 SAC3/sac3Δ::hphMX4 SPC110/SPC110-3XTEV696-HA::natMX4 ura3-1/GAL-NLS-myc9-TEVprotease-NLS2::URA3* | This Study |
| KGY246 | *MAT****a****/MATα ade3Δ/ade3Δ-100 CYH2^s^/cyh2^r^ LYS2/lys2Δ::HIS3 NUP60/nup60Δ::hphMX4 SPC110/SPC110-3XTEV696-HA::natMX4 ura3-1/GAL-NLS-myc9-TEVprotease-NLS2::URA3* | This Study |
| KGY247 | *MAT****a****/MATα ade3Δ/ade3Δ-100 CYH2^s^/cyh2^r^ LYS2/lys2Δ::HIS3 DYN3/dyn3Δ::hphMX4 SPC110/SPC110-3XTEV696-HA::natMX4 ura3-1/GAL-NLS-myc9-TEVprotease-NLS2::URA3* | This Study |
| KGY248 | *MAT****a****/MATα ade3Δ/ade3Δ-100 LYS2/lys2Δ::HIS3 KAR3/kar3Δ::hphMX4 SPC110/SPC110-3XTEV696-HA::natMX4 ura3-1/GAL-NLS-myc9-TEVprotease-NLS2::URA3* | This Study |
| KGY249 | *MAT****a****/MATα ade3Δ/ade3Δ-100 CYH2^s^/cyh2^r^ LYS2/lys2Δ::HIS3 HCM1/hcm1Δ::hphMX4 SPC110/SPC110-3XTEV696-HA::natMX4 ura3-1/GAL-NLS-myc9-TEVprotease-NLS2::URA3* | This Study |
| KGY250 | *MAT****a****/MATα ade3Δ/ade3Δ-100 LYS2/lys2Δ::HIS3 RTS1/rts1Δ::hphMX4 SPC110/SPC110-3XTEV696-HA::natMX4 ura3-1/GAL-NLS-myc9-TEVprotease-NLS2::URA3* | This Study |
| KGY252 | *MAT****a****/MATα ade3Δ/ade3Δ-100 LYS2/lys2Δ::HIS3 JNM1/jnm1Δ::hphMX4 SPC110/SPC110-3XTEV696-HA::natMX4 ura3-1/GAL-NLS-myc9-TEVprotease-NLS2::URA3* | This Study |
| KGY253 | *MAT****a****/MATα ade3Δ/ade3Δ-100 LYS2/lys2Δ::HIS3 DOT1/dot1Δ::hphMX4 SPC110/SPC110-3XTEV696-HA::natMX4 ura3-1/GAL-NLS-myc9-TEVprotease-NLS2::URA3* | This Study |
| KGY254 | *MAT****a****/MATα ade3Δ/ade3Δ-100 LYS2/lys2Δ::HIS3 POM152/pom152Δ::hphMX4 SPC110/SPC110-3XTEV696-HA::natMX4 ura3-1/GAL-NLS-myc9-TEVprotease-NLS2::URA3* | This Study |
| KGY255 | *MAT****a****/MATα ADE3/ade3Δ-100 CYH2^s^/cyh2^r^ LYS2/lys2Δ::HIS3 UBC4/ubc4Δ::hphMX4 SPC110/SPC110-3XTEV696-HA::natMX4 ura3-1/GAL-NLS-myc9-TEVprotease-NLS2::URA3* | This Study |
| KGY290-1D | *MATα ade3Δ cik1Δ::hphMX4* | This Study |
| KGY291-1A | *MATα ade3Δ vik1Δ::hphMX4* | This Study |
| KGY292-1A | *MATα ade3Δ mlp2Δ::hphMX4* | This Study |
| KGY302-7B | *MAT****a*** *ade3Δ SPC42::GFP-kanMX6* | This Study |
| KGY307-6B | *MAT****a*** *ade3Δ ubc4Δ::hphMX4 SPC42::GFP-kanMX6* | This Study |
| KGY308-1C | *MAT****a*** *cyh2^r^ sac3Δ::hphMX4 SPC110::GFP-kanMX6* | This Study |
| KGY309-1A | *MAT****a*** *nup60Δ::hphMX4 SPC110::GFP-kanMX6* | This Study |
| KGY311-5A | *MAT****a*** *ade3Δ jnm1Δ::hphMX4 SPC110::GFP-kanMX6* | This Study |
| KGY312-2D | *MAT****a*** *ade3Δ pom152Δ::hphMX4 SPC110::GFP-kanMX6* | This Study |
| KGY313-3B | *MAT****a*** *cyh2^r^ ubc4Δ::hphMX4 SPC110::GFP-kanMX6* | This Study |
| KGY315 | *MAT****a****/MATα ADE3/ade3Δ CYH2^s^/cyh2^r^* | This Study |
| KGY321-3A | *MATα ade3Δ-100 cyh2^r^ lys2Δ::HIS3 GAL-NLS-myc9-TEVprotease-NLS2::URA3* | This Study |
| KGY364-1A | *MATα ppm1Δ::hphMX4* | This Study |
| KGY373-4D | *MAT****a*** *nup60Δ::hphMX4 SPC110::GFP-kanMX6 GAL-myc-MPS1::URA3* | This Study |
| KGY375-7C | *MAT****a*** *jnm1Δ::hphMX4 SPC110::GFP-kanMX6 GAL-myc-MPS1::URA3* | This Study |
| KGY376-8D | *MAT****a*** *pom152Δ::hphMX4 SPC110::GFP-kanMX6 GAL-myc-MPS1::URA3* | This Study |
| KGY377-6D | *MAT****a*** *ubc4Δ::hphMX4 SPC110::GFP-kanMX6 GAL-myc-MPS1::URA3* | This Study |
| KGY378-1D | *MAT****a*** *SPC110::GFP-kanMX6 GAL-myc-MPS1::URA3* | This Study |
| KGY379 | *MAT****a****/MATα ade3Δ/ade3Δ HIS3/his3-11,15 CIK1/cik1Δ::hphMX4 SPC110/spc110-220* | This Study |
| KGY380 | *MAT****a****/MATα ade3Δ/ade3Δ HIS3/his3-11,15 MLP2/mlp2Δ::hphMX4 SPC110/spc110-220* | This Study |
| KGY381 | *MAT****a****/MATα ade3Δ/ade3Δ HIS3/his3-11,15 VIK1/vik1Δ::hphMX4 SPC110/spc110-220* | This Study |
| KGY382 | *MAT****a****/MATα ADE3/ade3Δ HIS3/his3-11,15 PPM1/ppm1Δ::hphMX4 SPC110/spc110-220* | This Study |
| KGY383 | *MAT****a****/MATα ade3Δ/ade3Δ CIK1/cik1Δ::hphMX4 SPC110/spc110-221* | This Study |
| KGY384 | *MAT****a****/MATα ade3Δ/ade3Δ MLP2/mlp2Δ::hphMX4 SPC110/spc110-221* | This Study |
| KGY385 | *MAT****a****/MATα ade3Δ/ade3Δ VIK1/vik1Δ::hphMX4 SPC110/spc110-221* | This Study |
| KGY386 | *MAT****a****/MATα ADE3/ade3Δ PPM1/ppm1Δ::hphMX4 SPC110/spc110-221* | This Study |
| KGY387 | *MAT****a****/MATα ade3Δ/ade3Δ LYS2/lys2Δ::HIS3 CIK1/cik1Δ::hphMX4 SPC110/spc110-226* | This Study |
| KGY388 | *MAT****a****/MATα ade3Δ/ade3Δ LYS2/lys2Δ::HIS3 MLP2/mlp2Δ::hphMX4 SPC110/spc110-226* | This Study |
| KGY389 | *MAT****a****/MATα ade3Δ/ade3Δ LYS2/lys2Δ::HIS3 VIK1/vik1Δ::hphMX4 SPC110/spc110-226* | This Study |
| KGY390 | *MAT****a****/MATα ADE3/ade3Δ LYS2/lys2Δ::HIS3 PPM1/ppm1Δ::hphMX4 SPC110/spc110-226* | This Study |
| KGY391 | *MAT****a****/MATα ade3Δ/ade3Δ-100 CYH2^s^/cyh2^r^ LYS2/lys2Δ::HIS3 CIK1/cik1Δ::hphMX4 SPC110/SPC110-3XTEV696-HA::natMX4 ura3-1/GAL-NLS-myc9-TEVprotease-NLS2::URA3* | This Study |
| KGY392 | *MAT****a****/MATα ade3Δ/ade3Δ-100 CYH2^s^/cyh2^r^ LYS2/lys2Δ::HIS3 MLP2/mlp2Δ::hphMX4 SPC110/SPC110-3XTEV696-HA::natMX4 ura3-1/GAL-NLS-myc9-TEVprotease-NLS2::URA3* | This Study |
| KGY393 | *MAT****a****/MATα ade3Δ/ade3Δ-100 CYH2^s^/cyh2^r^ LYS2/lys2Δ::HIS3 VIK1/vik1Δ::hphMX4 SPC110/SPC110-3XTEV696-HA::natMX4 ura3-1/GAL-NLS-myc9-TEVprotease-NLS2::URA3* | This Study |
| KGY394 | *MAT****a****/MATα ADE3/ade3Δ-100 CYH2^s^/cyh2^r^ LYS2/lys2Δ::HIS3 PPM1/ppm1Δ::hphMX4 SPC110/SPC110-3XTEV696-HA::natMX4 ura3-1/GAL-NLS-myc9-TEVprotease-NLS2::URA3* | This Study |
| KGY399-1A | *MAT****a*** *mlp2Δ::hphMX4 SPC110::GFP-kanMX6* | This Study |
| KGY405-2C | *MATα ncs2Δ::hphMX4* | This Study |
| KGY406-1B | *MATα lsm7Δ::hphMX4* | This Study |
| KGY409 | *MAT****a****/MATα ADE3/ade3Δ HIS3/his3-11,15 NCS2/ncs2Δ::hphMX4 SPC110/spc110-220* | This Study |
| KGY410 | *MAT****a****/MATα ADE3/ade3Δ NCS2/ncs2Δ::hphMX4 SPC110/spc110-221* | This Study |
| KGY411 | *MAT****a****/MATα ADE3/ade3Δ LYS2/lys2Δ::HIS3 NCS2/ncs2Δ::hphMX4 SPC110/spc110-226* | This Study |
| KGY412 | *MAT****a****/MATα ADE3/ade3Δ-100 CYH2^s^/cyh2^r^ LYS2/lys2Δ::HIS3 NCS2/ncs2Δ::hphMX4 SPC110/SPC110-3XTEV696-HA::natMX4 ura3-1/GAL-NLS-myc9-TEVprotease-NLS2::URA3* | This Study |
| KGY414-2B | *MAT****a*** *ncs2Δ::hphMX4 SPC110::GFP-kanMX6* | This Study |
| KGY415 | *MAT****a****/MATα ADE3/ade3Δ HIS3/his3-11,15 LSM7/lsm7Δ::hphMX4 SPC110/spc110-220* | This Study |
| KGY416 | *MAT****a****/MATα ADE3/ade3Δ LSM7/lsm7Δ::hphMX4 SPC110/spc110-221* | This Study |
| KGY417 | *MAT****a****/MATα ADE3/ade3Δ LYS2/lys2Δ::HIS3 LSM7/lsm7Δ::hphMX4 SPC110/spc110-226* | This Study |
| KGY418 | *MAT****a****/MATα ADE3/ade3Δ-100 CYH2^s^/cyh2^r^ LYS2/lys2Δ::HIS3 LSM7/lsm7Δ::hphMX4 SPC110/SPC110-3XTEV696-HA::natMX4 ura3-1/GAL-NLS-myc9-TEVprotease-NLS2::URA3* | This Study |
| KGY428-4D | *MAT****a*** *mlp2Δ::hphMX4 SPC110::GFP-kanMX6 GAL-myc-MPS1::URA3* | This Study |
| KGY429-7C | *MAT****a*** *ppm1Δ::hphMX4 SPC110::GFP-kanMX6 GAL-myc-MPS1::URA3* | This Study |
| KGY431-12B | *MAT****a*** *ncs2Δ::hphMX4 SPC110::GFP-kanMX6 GAL-myc-MPS1::URA3* | This Study |
| KGY446 | *MAT****a****/MATα ADE3/ade3Δ-100 CYH2^s^/cyh2^r^ ubc5Δ::kanMX6/UBC5 SPC110/spc110-220* | This Study |
| KGY447 | *MAT****a****/MATα ADE3/ade3Δ-100 CYH2^s^/cyh2^r^ ubc5Δ::kanMX6/UBC5 SPC110/spc110-221* | This Study |
| KGY448 | *MAT****a****/MATα ade3Δ/ade3Δ CYH2^s^/cyh2^r^ LYS2/lys2Δ::HIS3 ubc5Δ::kanMX6/UBC5 SPC110/spc110-226* | This Study |
| KGY449-6B | *MAT****a*** *ade3Δ-100 cyh2^r^ ubc5Δ::kanMX6 SPC42::GFP-kanMX6* | This Study |
| KGY450-7C | *MAT****a*** *ade3Δ-100 ubc5Δ::kanMX6 SPC110::GFP-kanMX6* | This Study |
| KGY454-11D | *MAT****a*** *ubc5Δ::kanMX6 SPC110::GFP-kanMX6 GAL-myc-MPS1::URA3* | This Study |
| KGY457 | *MAT****a****/MATα ade3Δ-100/ade3Δ-100 CYH2^s^/cyh2^r^ LYS2/lys2Δ::HIS3 ubc5Δ::kanMX6/UBC5 ura3-1/GAL-NLS-myc9-TEVprotease-NLS2::URA3 SPC110/SPC110-3XTEV696-HA::natMX4* | This Study |
| KSY2-2C | *MAT****a*** *ubc4Δ::hphMX4* | Kate Stoll |
| KSY7-1C | *MAT****a*** *ade3Δ-100 ubc5Δ::kanMX6* | Kate Stoll |
| SFY1 | *MAT****a*** *ade3Δ cyh2^r^ lys2Δ::HIS3 spc110-226 pHS26* | [5] |
| SFY2 | *MATα ade3Δ lys2Δ::HIS3 spc110-226 pHS26* | [5] |
| SFY127-1A | *MATα mad2Δ::kanMX6* | Susan Francis |
| TDY439-1B | *MAT****a*** *mad1Δ::URA3* | This Study |
| Y7029 | *MATα can1Δ::STE2pr-HIS3 lyp1Δ his3Δ1 leu2Δ0 ura3Δ0 met15Δ0 LYS2 (S288C background)* | [6] |
| W303 | *MAT****a*** *ade2-1oc can1-100 his3-11,15 leu2-3,112 trp1-1 ura3-1* |  |
| S288C | *MATα SUC2 gal2 mal mel flo1 flo8-1 hap1* |  |

^a^All strains have the same markers as W303 except as shown.

References

1. Hazbun TR, Malmstrom L, Anderson S, Graczyk BJ, Fox B, et al. (2003) Assigning function to yeast proteins by integration of technologies. Mol Cell 12: 1353-1365.

2. Geiser JR, Sundberg HA, Chang BH, Muller EG, Davis TN (1993) The essential mitotic target of calmodulin is the 110-kilodalton component of the spindle pole body in Saccharomyces cerevisiae. Mol Cell Biol 13: 7913-7924.

3. Sundberg HA, Goetsch L, Byers B, Davis TN (1996) Role of calmodulin and Spc110p interaction in the proper assembly of spindle pole body compenents. J Cell Biol 133: 111-124.

4. Sundberg HA, Davis TN (1997) A mutational analysis identifies three functional regions of the spindle pole component Spc110p in Saccharomyces cerevisiae. Mol Biol Cell 8: 2575-2590.

5. Shimogawa MM, Graczyk B, Gardner MK, Francis SE, White EA, et al. (2006) Mps1 phosphorylation of Dam1 couples kinetochores to microtubule plus ends at metaphase. Curr Biol 16: 1489-1501.

6. Tong AH, Boone C (2006) Synthetic genetic array analysis in Saccharomyces cerevisiae. Methods Mol Biol 313: 171-192.
