# Supplementary figures and images for: Identification of Saccharomyces cerevisiae Spindle Pole Body Remodeling Factors
Source: PLoS One. 2010 Nov 12;5(11):e15426. doi: 10.1371/journal.pone.0015426 (PMC2980476; doi:10.1371/journal.pone.0015426)

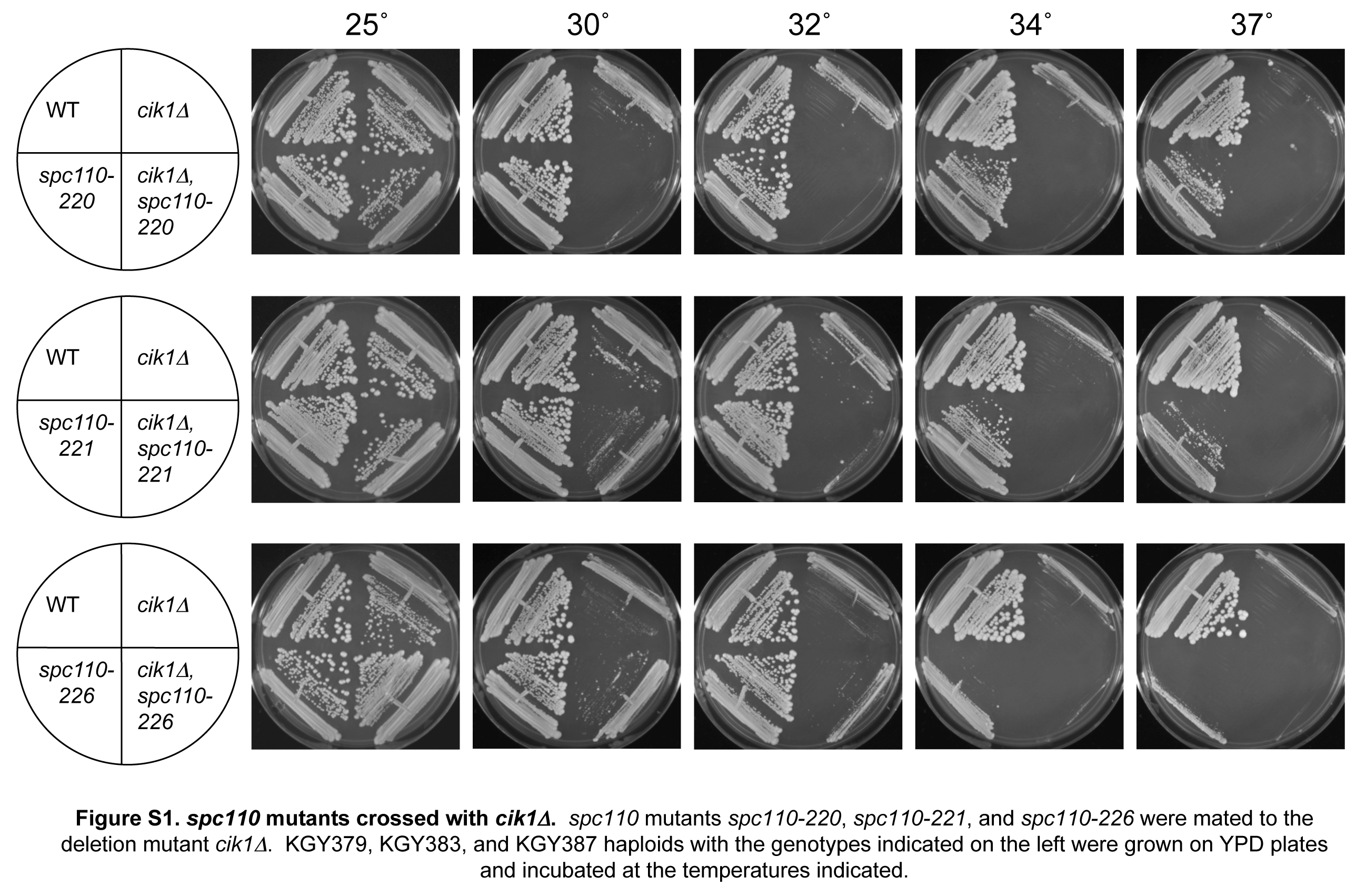

Supplement: Figure S1 — spc110 mutants crossed with cik1 . Haploids with the genotypes indicated on the left were grown on YPD plates and incubated at the temperatures indicated. (TIF) [file pone.0015426.s004.tif]

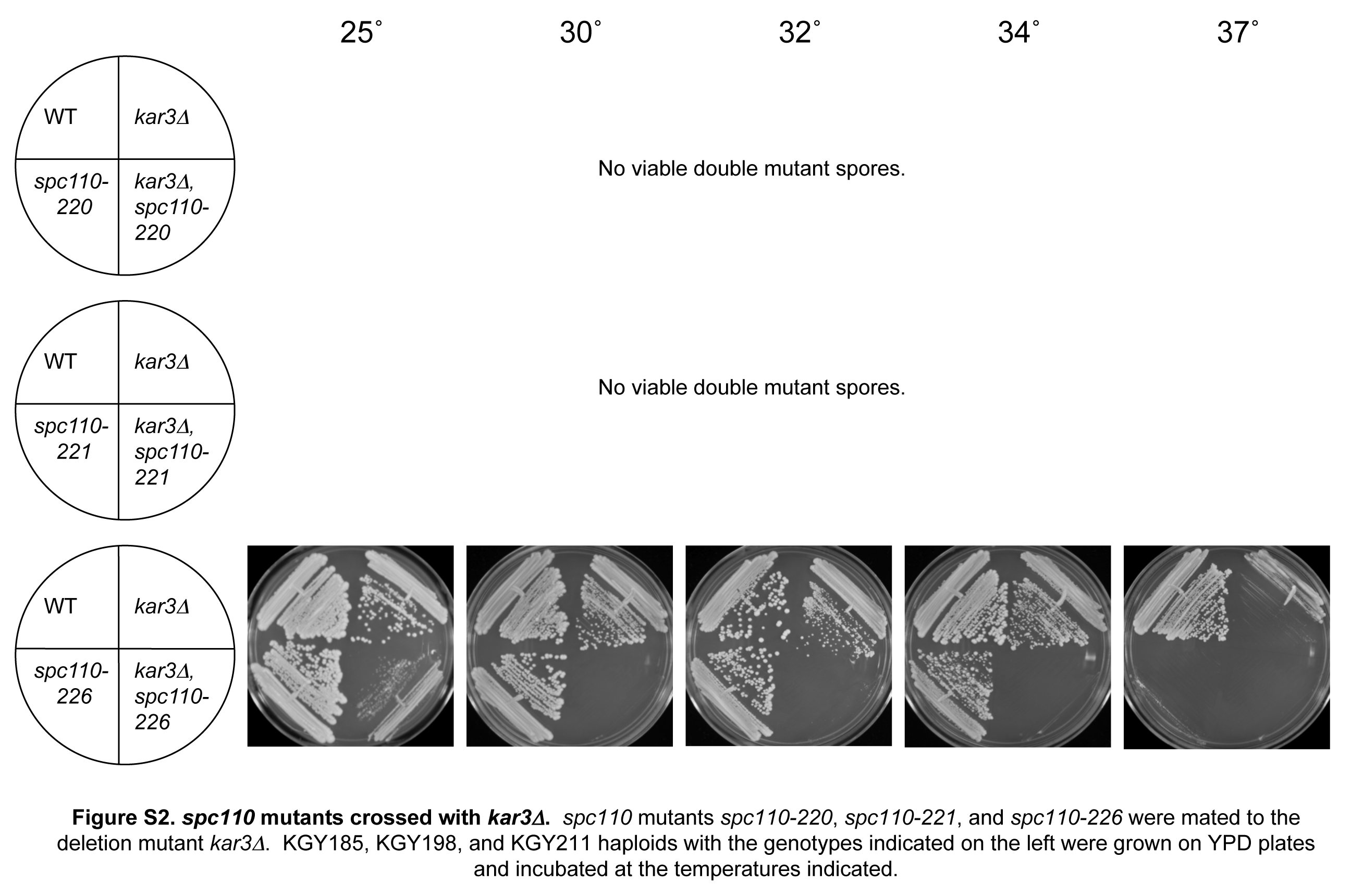

Supplement: Figure S2 — spc110 mutants crossed with kar3 . Haploids with the genotypes indicated on the left were grown on YPD plates and incubated at the temperatures indicated. (TIF) [file pone.0015426.s005.tif]

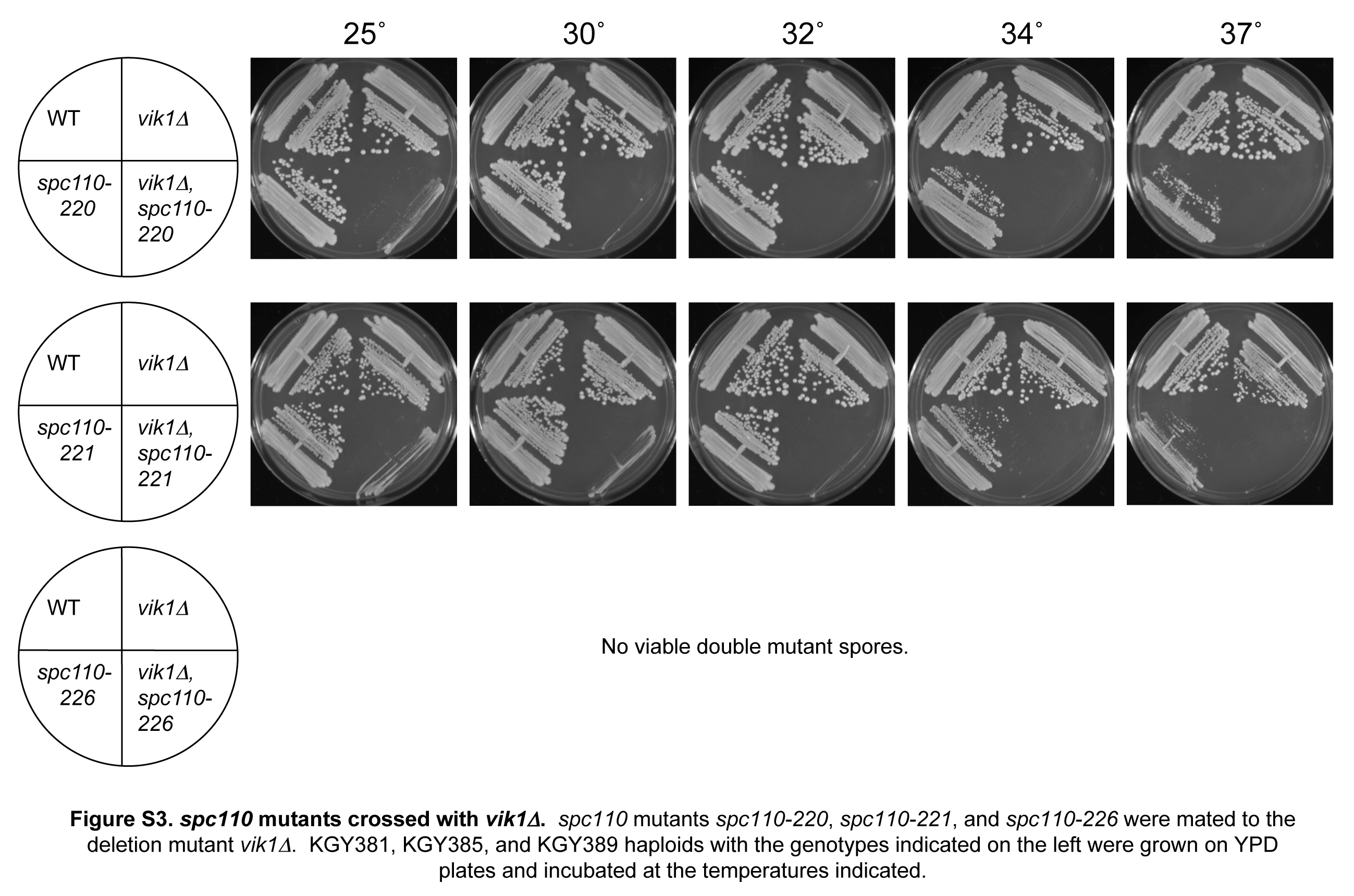

Supplement: Figure S3 — spc110 mutants crossed with vik1 . Haploids with the genotypes indicated on the left were grown on YPD plates and incubated at the temperatures indicated. (TIF) [file pone.0015426.s006.tif]

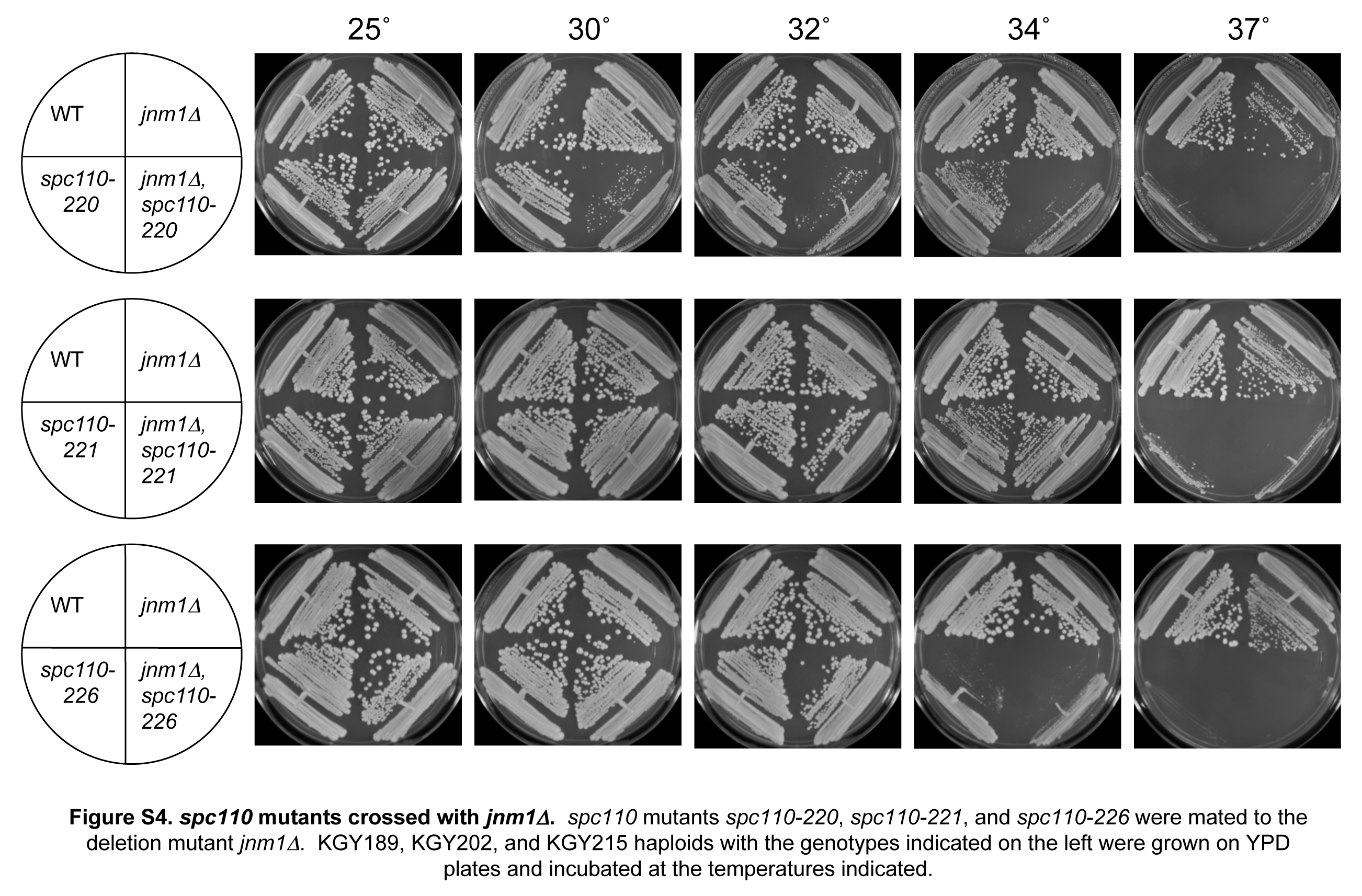

Supplement: Figure S4 — spc110 mutants crossed with jnm1 . Haploids with the genotypes indicated on the left were grown on YPD plates and incubated at the temperatures indicated. (TIF) [file pone.0015426.s007.tif]

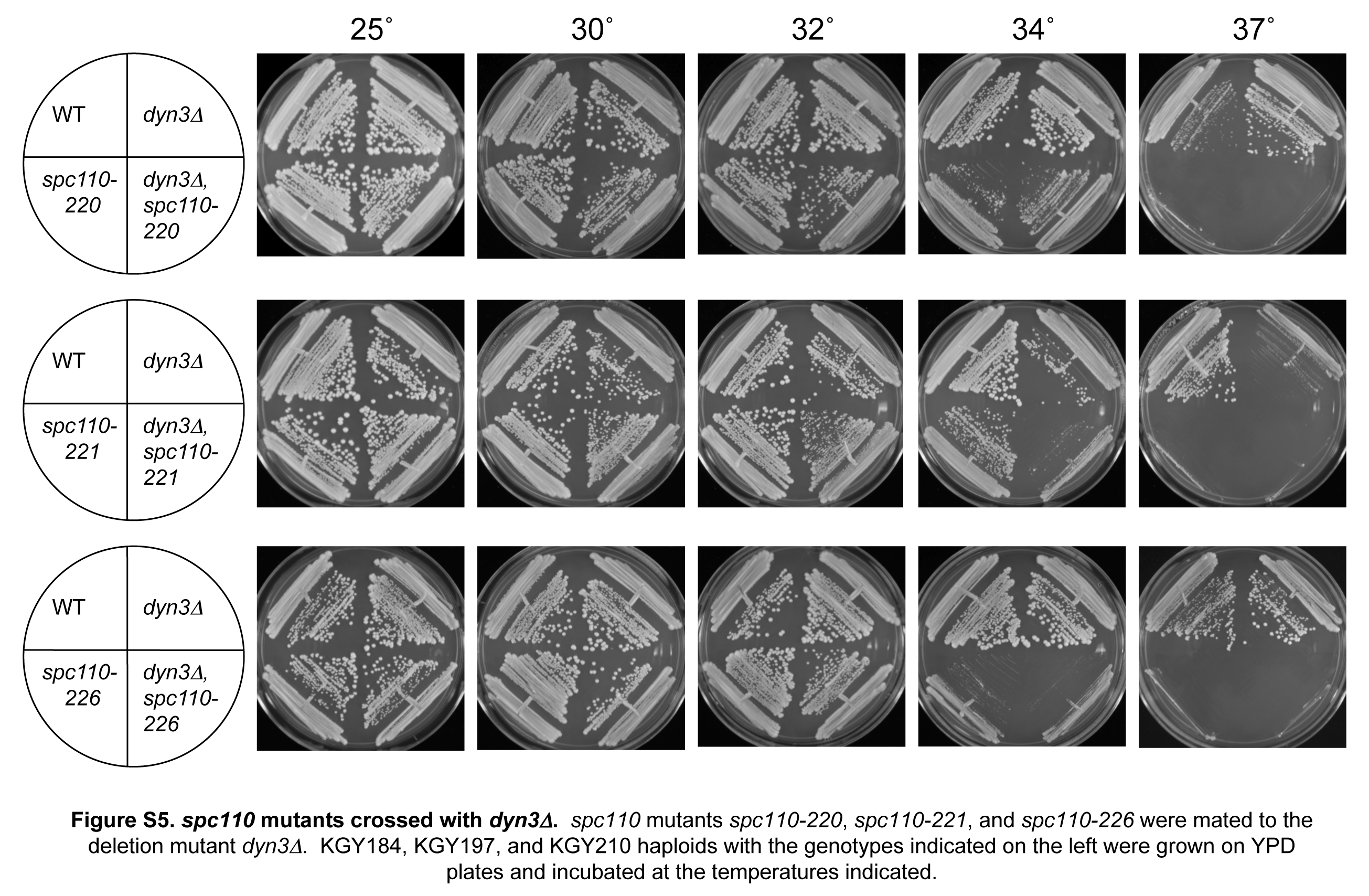

Supplement: Figure S5 — spc110 mutants crossed with dyn3 . Haploids with the genotypes indicated on the left were grown on YPD plates and incubated at the temperatures indicated. (TIF) [file pone.0015426.s008.tif]

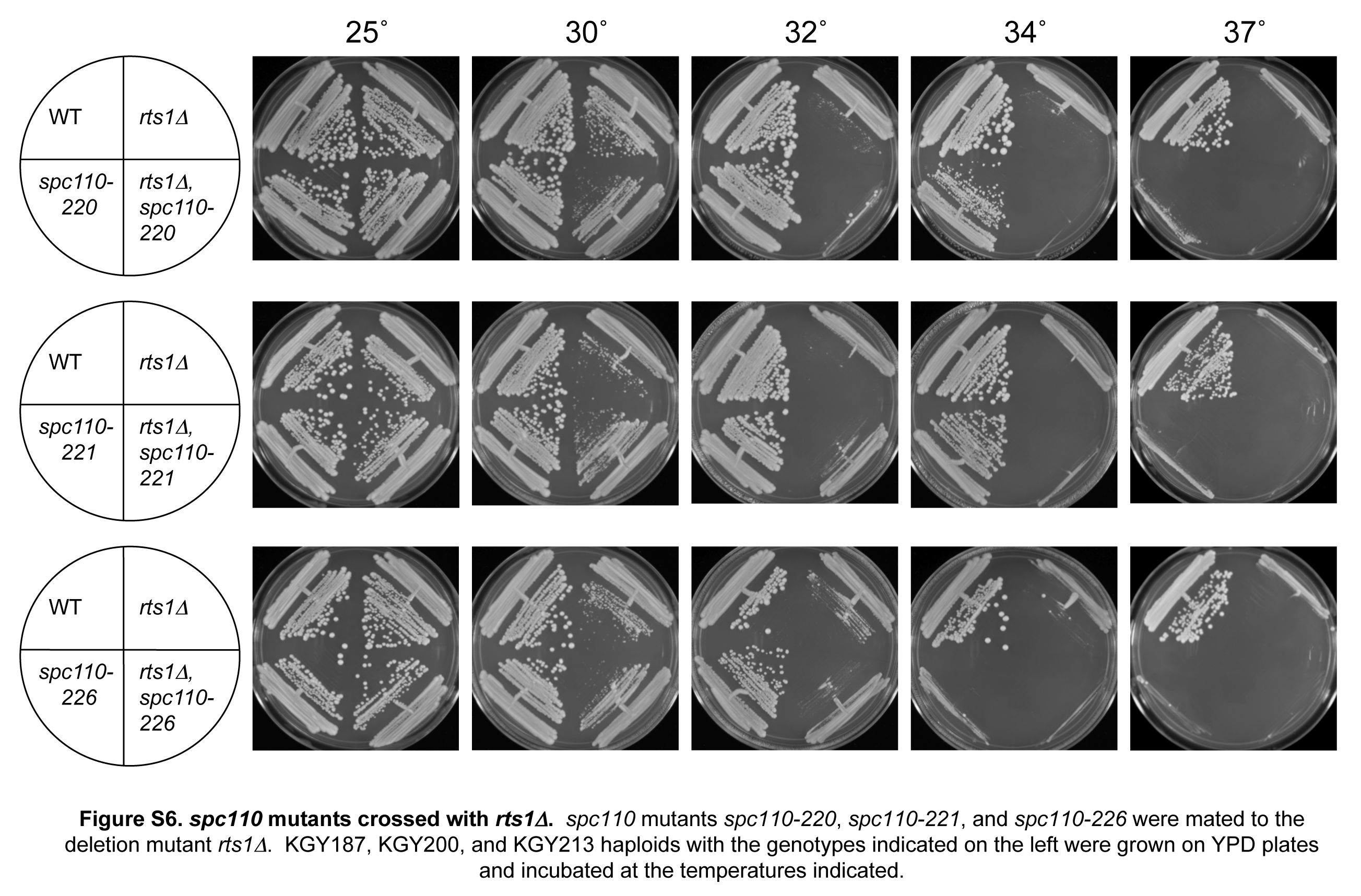

Supplement: Figure S6 — spc110 mutants crossed with rts1 . Haploids with the genotypes indicated on the left were grown on YPD plates and incubated at the temperatures indicated. (TIF) [file pone.0015426.s009.tif]

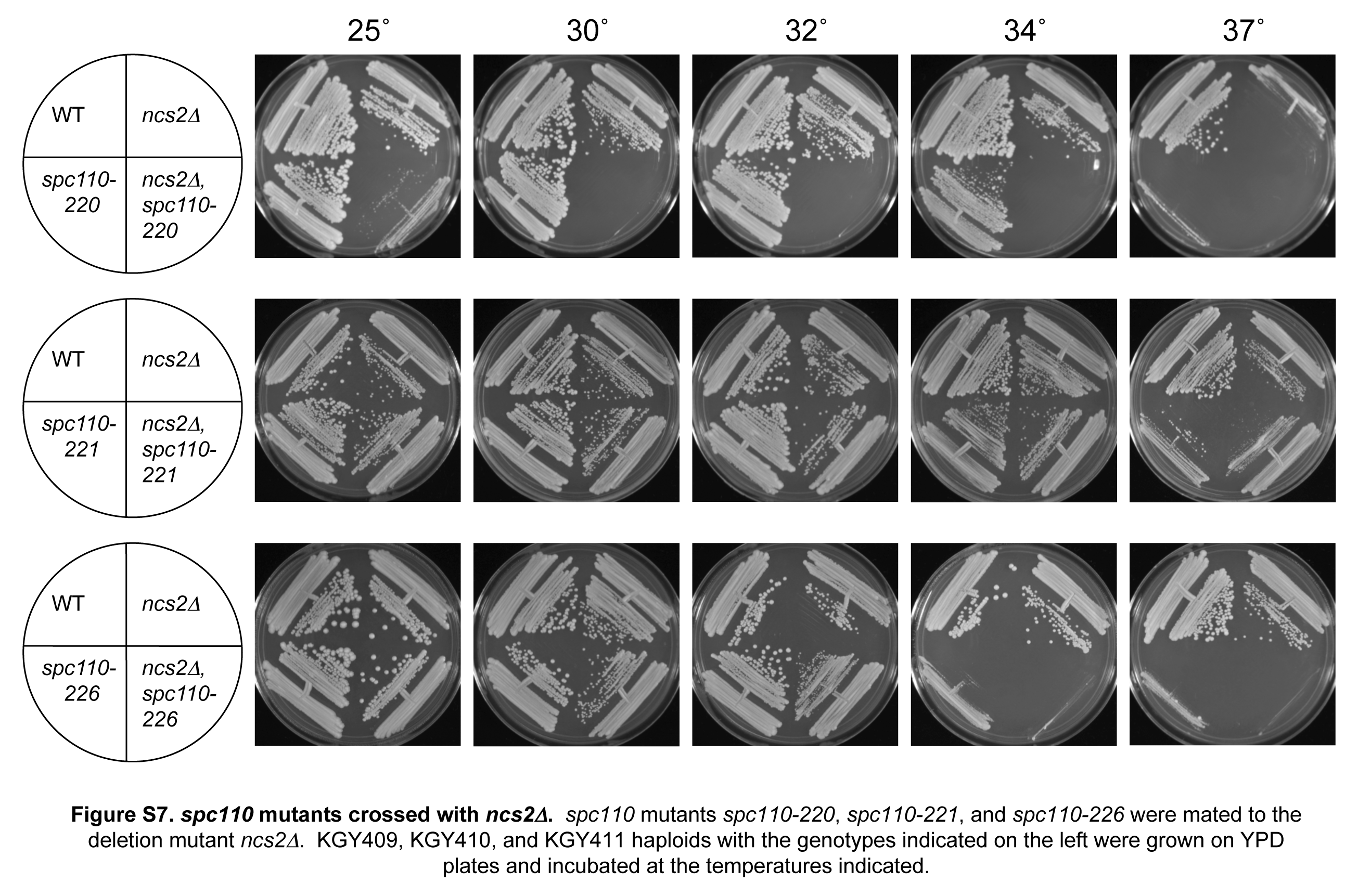

Supplement: Figure S7 — spc110 mutants crossed with ncs2 . Haploids with the genotypes indicated on the left were grown on YPD plates and incubated at the temperatures indicated. (TIF) [file pone.0015426.s010.tif]

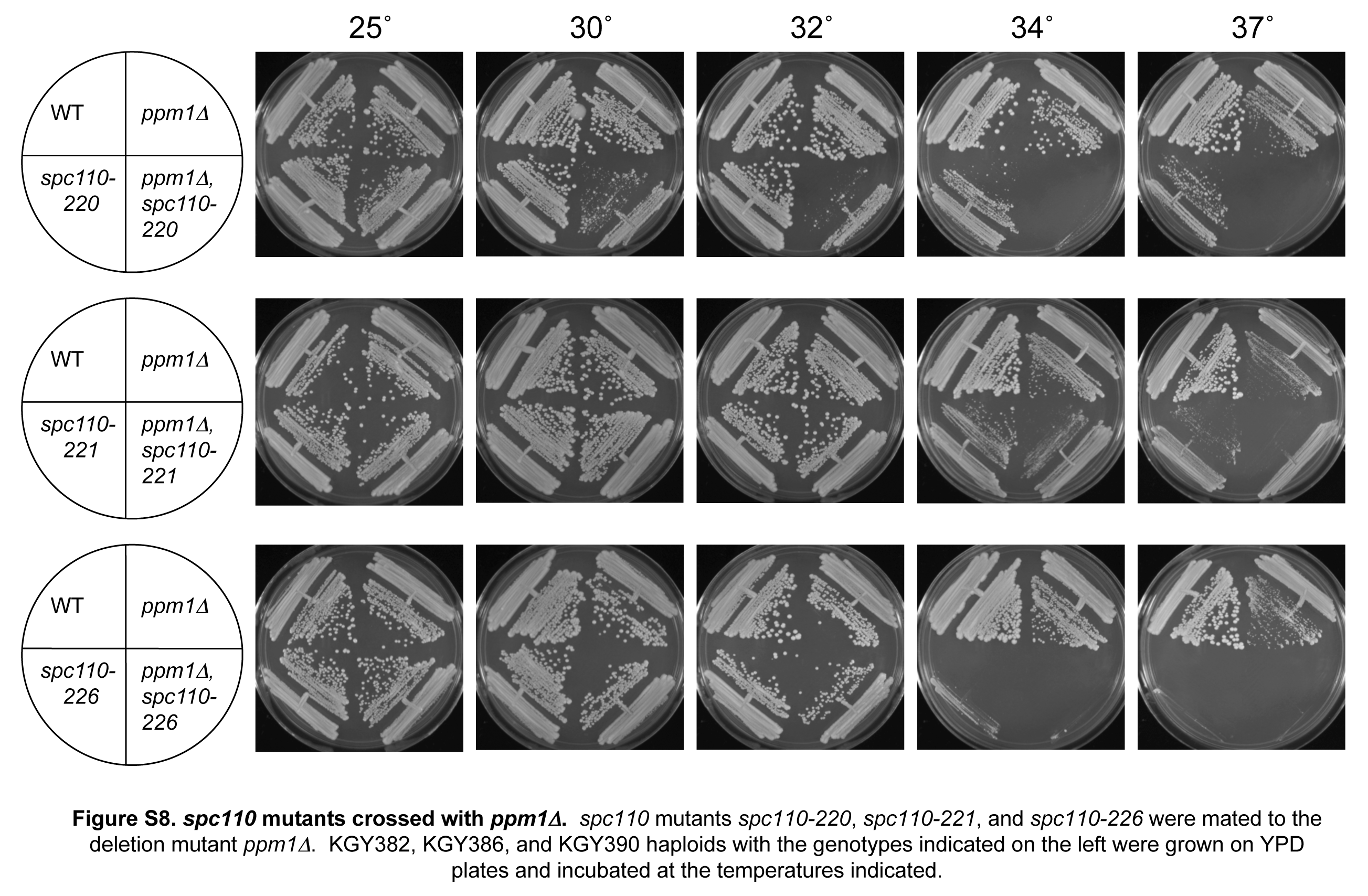

Supplement: Figure S8 — spc110 mutants crossed with ppm1 . Haploids with the genotypes indicated on the left were grown on YPD plates and incubated at the temperatures indicated. (TIF) [file pone.0015426.s011.tif]

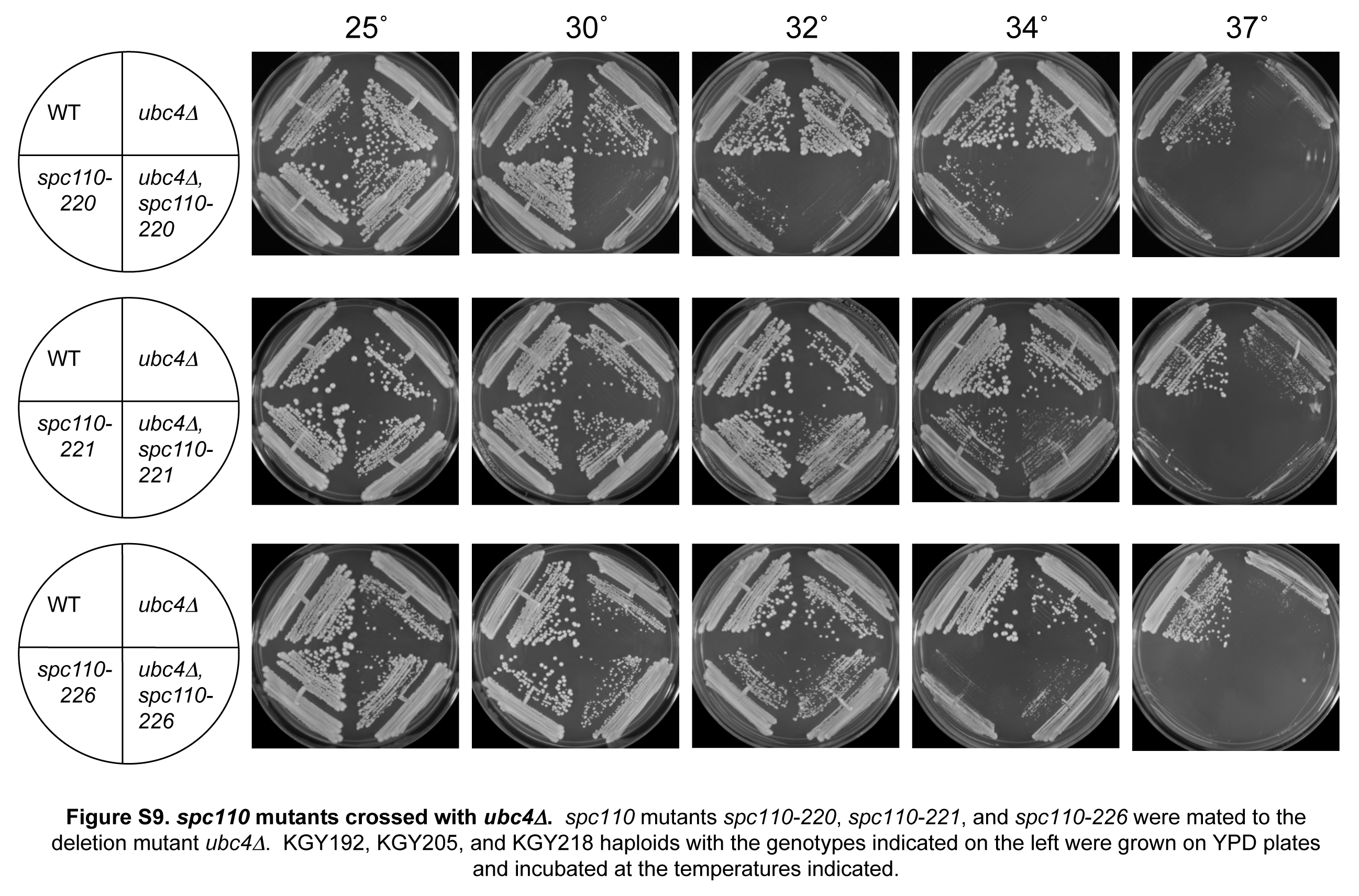

Supplement: Figure S9 — spc110 mutants crossed with ubc4 . Haploids with the genotypes indicated on the left were grown on YPD plates and incubated at the temperatures indicated. (TIF) [file pone.0015426.s012.tif]

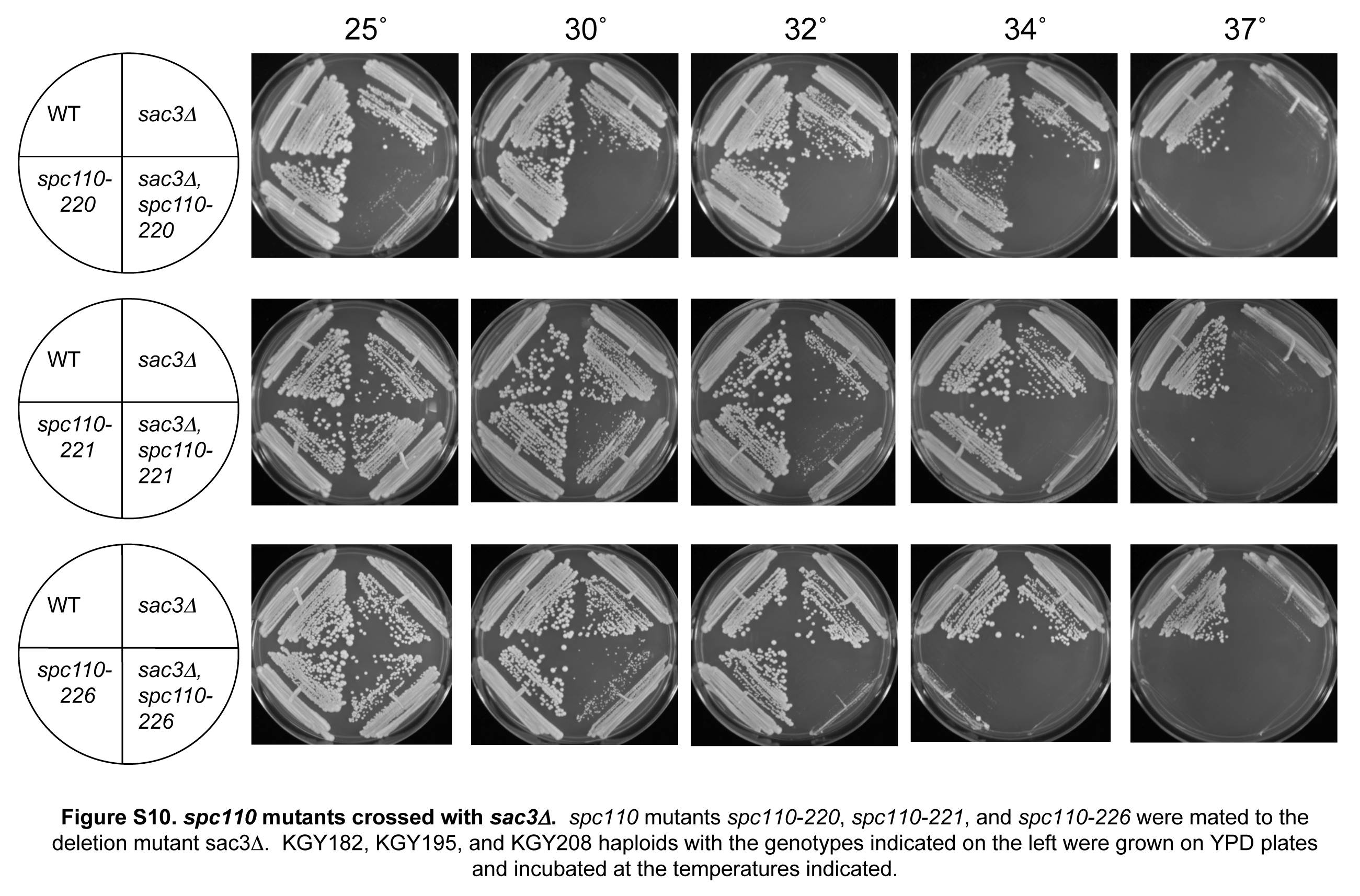

Supplement: Figure S10 — spc110 mutants crossed with sac3 . Haploids with the genotypes indicated on the left were grown on YPD plates and incubated at the temperatures indicated. (TIF) [file pone.0015426.s013.tif]

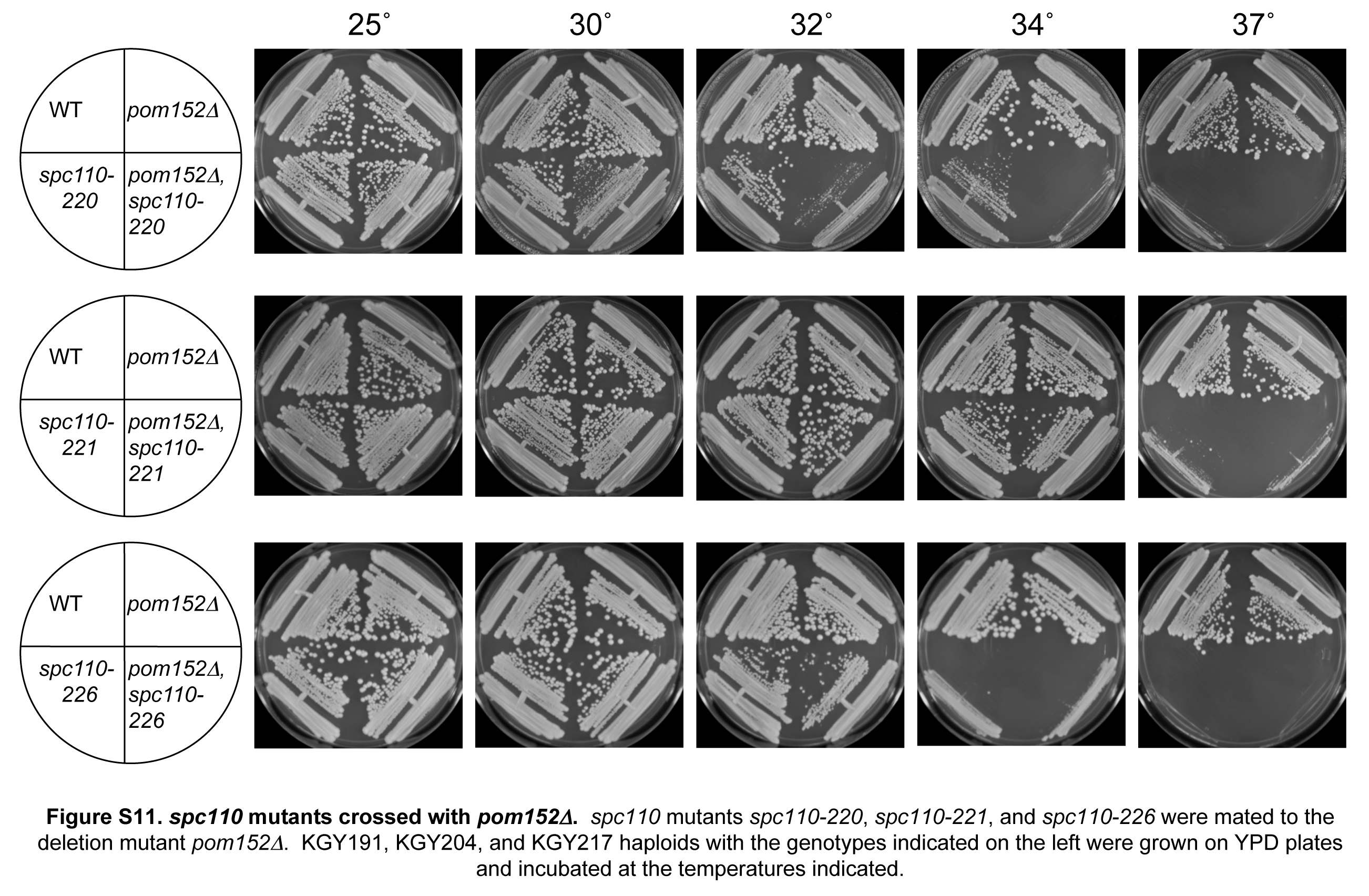

Supplement: Figure S11 — spc110 mutants crossed with pom152 . Haploids with the genotypes indicated on the left were grown on YPD plates and incubated at the temperatures indicated. (TIF) [file pone.0015426.s014.tif]

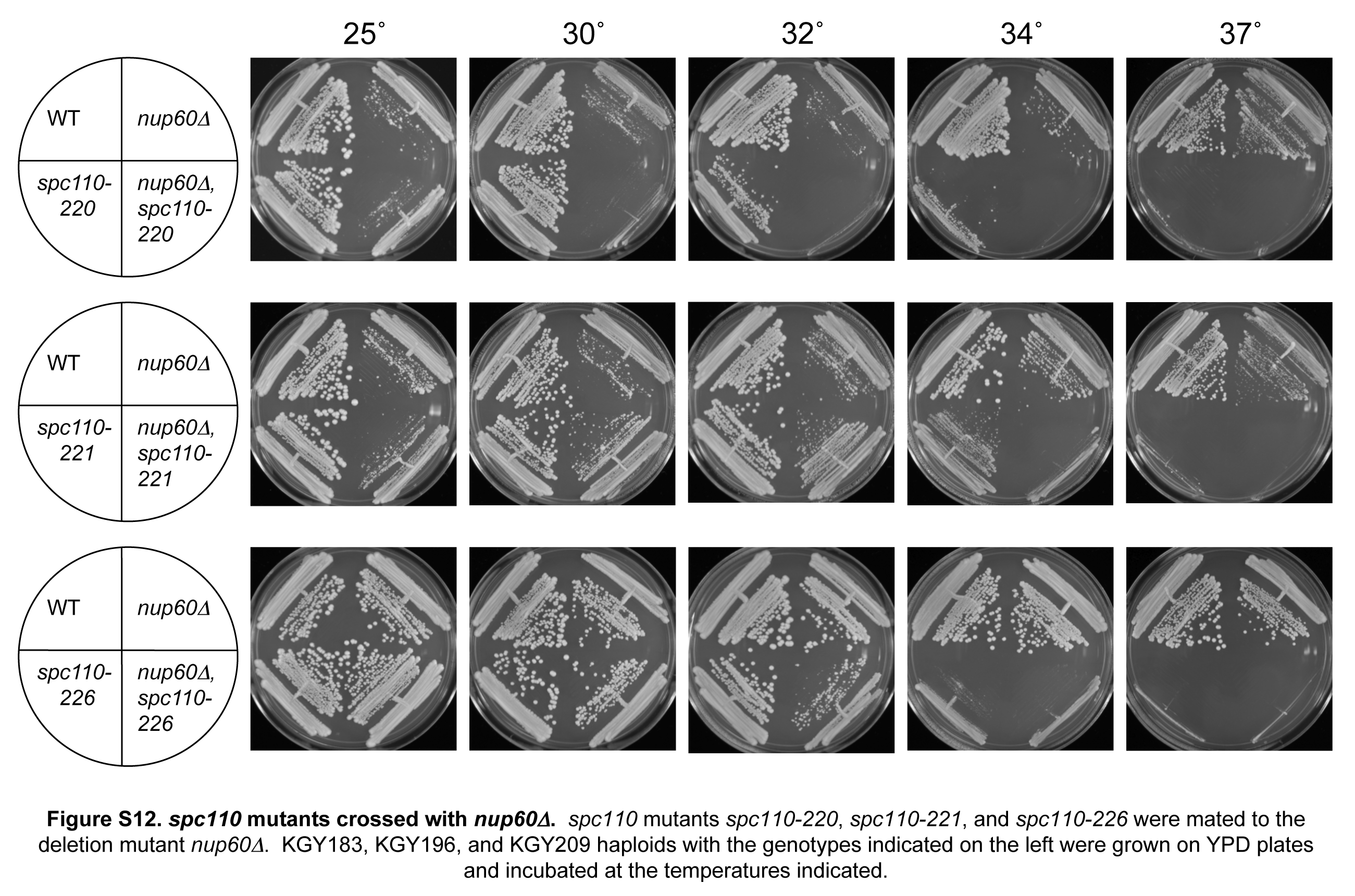

Supplement: Figure S12 — spc110 mutants crossed with nup60 . Haploids with the genotypes indicated on the left were grown on YPD plates and incubated at the temperatures indicated. (TIF) [file pone.0015426.s015.tif]

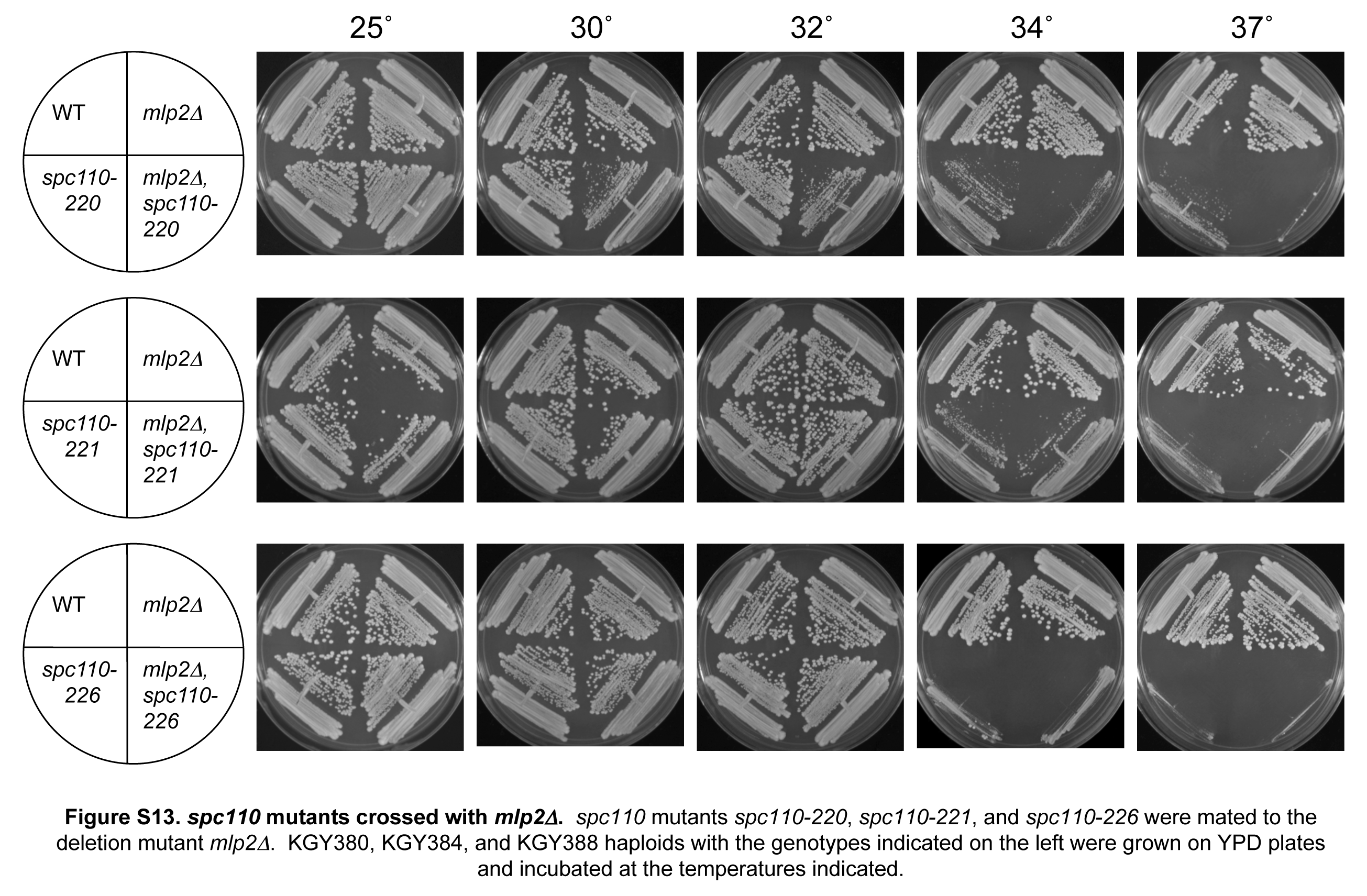

Supplement: Figure S13 — spc110 mutants crossed with mlp2 . Haploids with the genotypes indicated on the left were grown on YPD plates and incubated at the temperatures indicated. (TIF) [file pone.0015426.s016.tif]

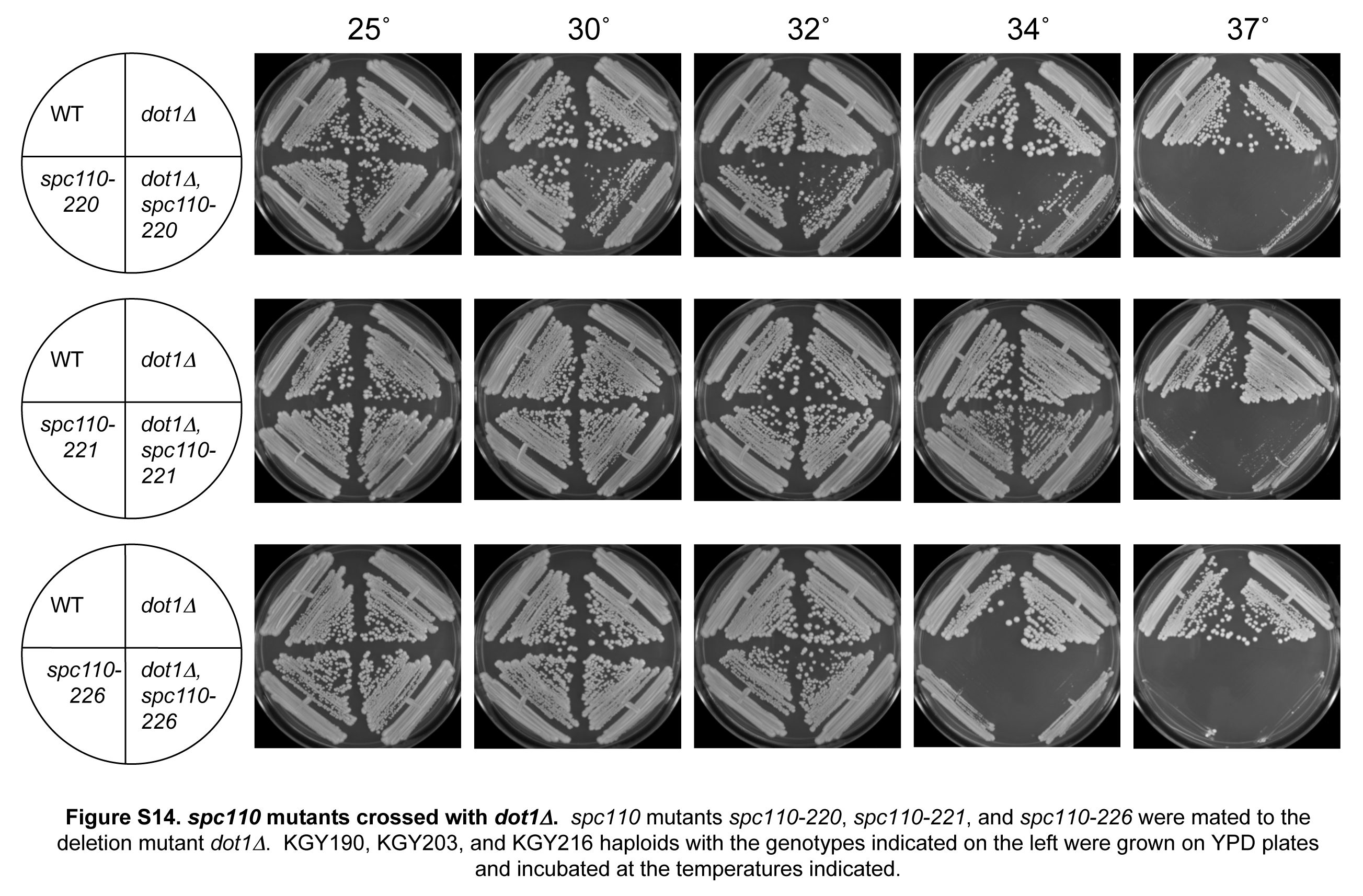

Supplement: Figure S14 — spc110 mutants crossed with dot1 . Haploids with the genotypes indicated on the left were grown on YPD plates and incubated at the temperatures indicated. (TIF) [file pone.0015426.s017.tif]

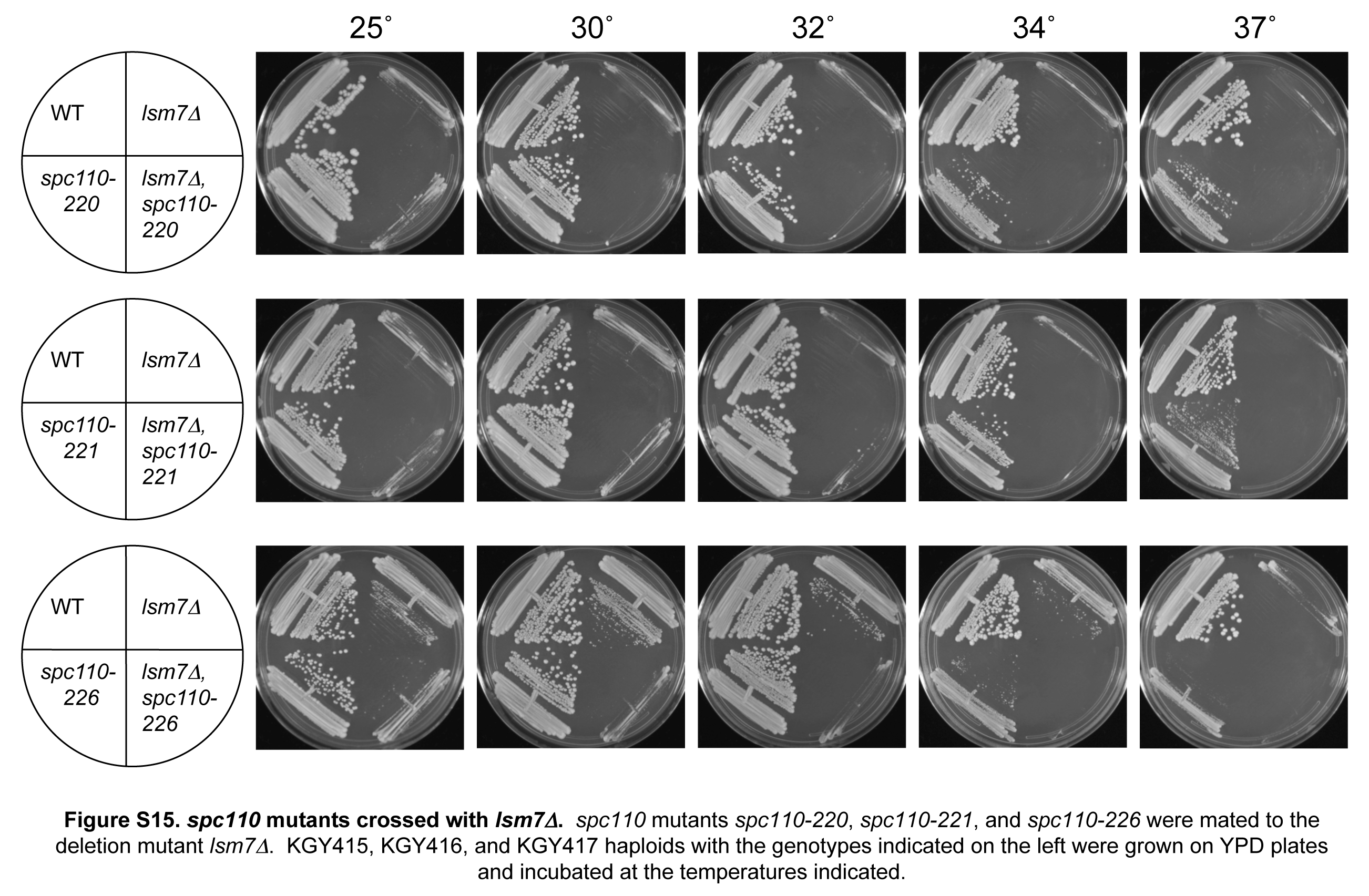

Supplement: Figure S15 — spc110 mutants crossed with lsm7 . Haploids with the genotypes indicated on the left were grown on YPD plates and incubated at the temperatures indicated. (TIF) [file pone.0015426.s018.tif]

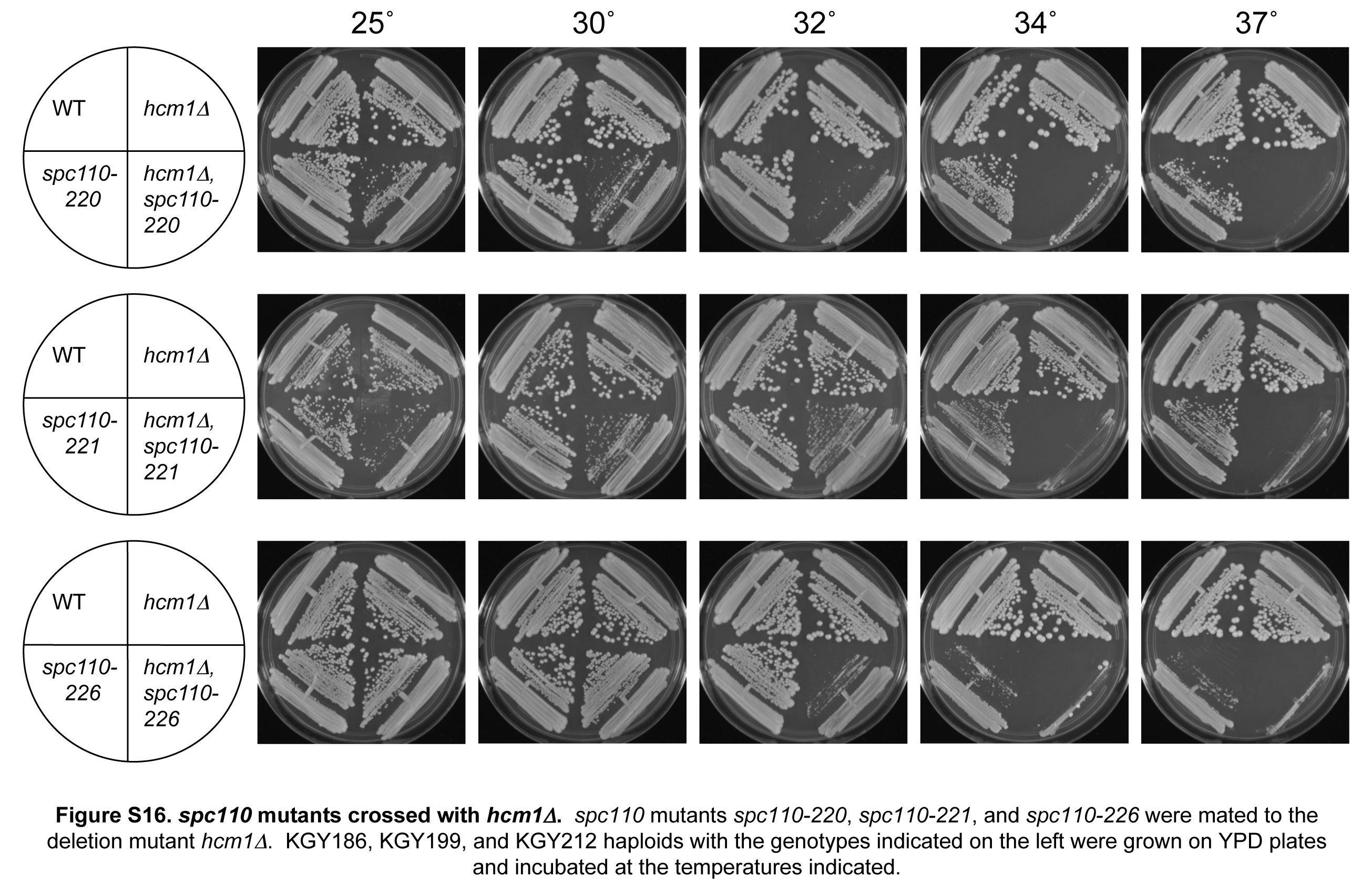

Supplement: Figure S16 — spc110 mutants crossed with hcm1 . Haploids with the genotypes indicated on the left were grown on YPD plates and incubated at the temperatures indicated. (TIF) [file pone.0015426.s019.tif]
